# Supplementary figures and images for: Sample size calculation for phylogenetic case linkage
Source: PLoS Comput Biol. 2021 Jul 6;17(7):e1009182. doi: 10.1371/journal.pcbi.1009182 (PMC8284614; doi:10.1371/journal.pcbi.1009182)

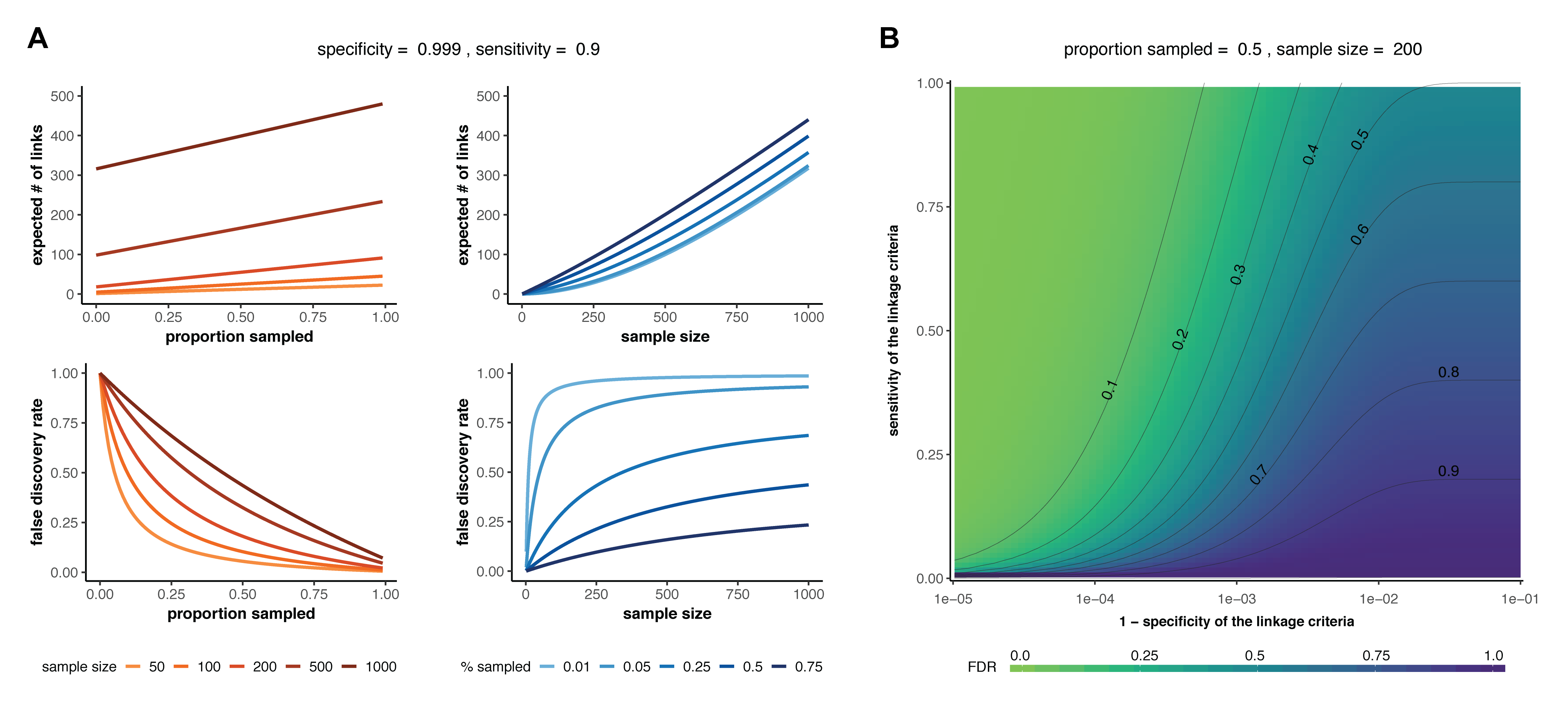

Supplement: S1 Fig — (A) Effect of sample size (red lines) or proportion sampled (blue lines) on the expected number of linked pairs (upper plots) or the false discovery rate of linked pairs (lower plots). The specificity and sensitivity are held constant. (B) Effect of varying the sensitivity and specificity of the linkage criteria on the false discovery rate (FDR). (TIF) [file pcbi.1009182.s001.tif]

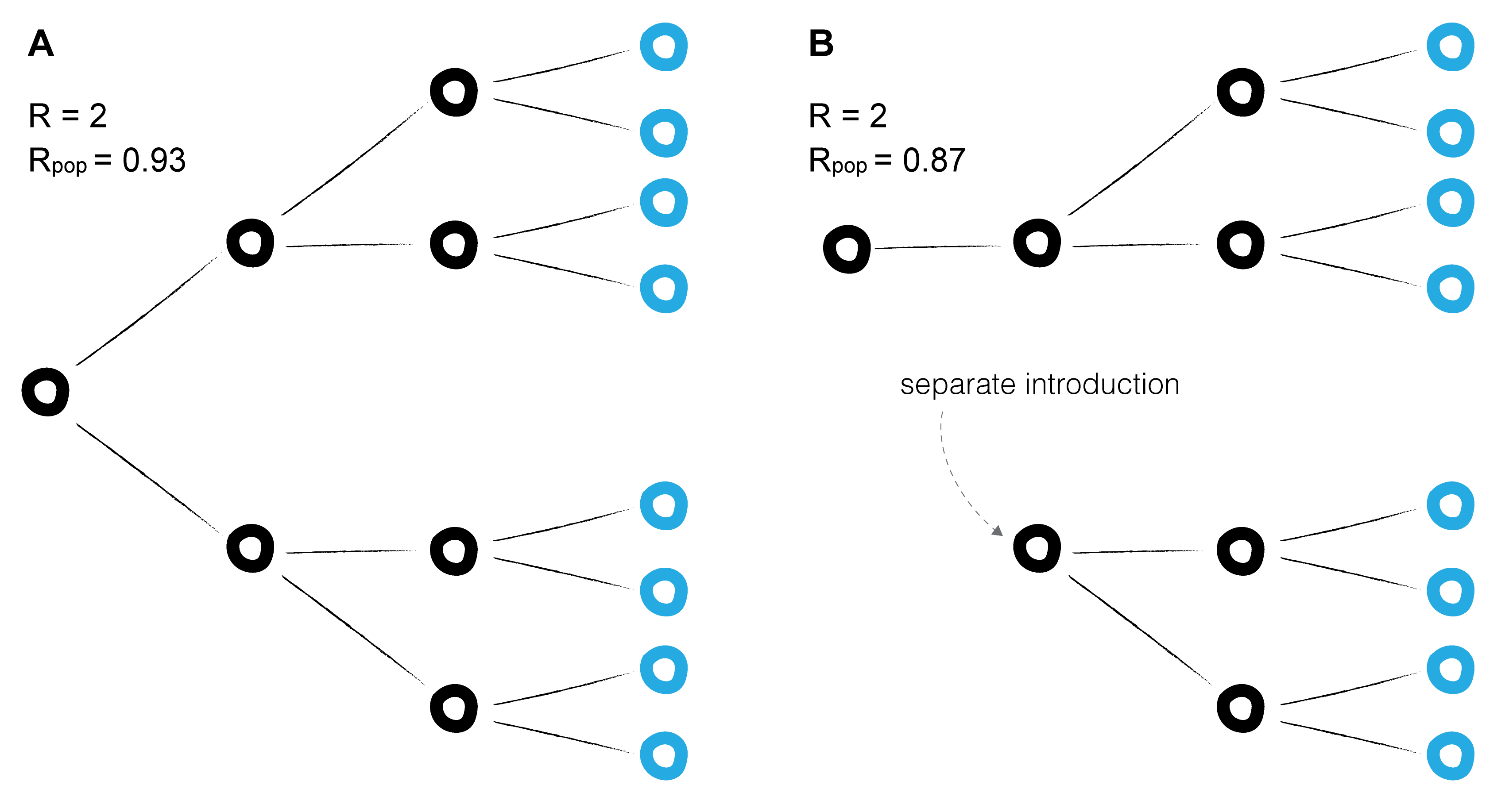

Supplement: S2 Fig — Two hypothetical outbreaks with a pathogen reproductive number (R) equal to 2 and a total of 15 infections. Black circles represent infections; blue circles represent infections who have not yet infected others, or whose descendents are outside the sampling frame. (A) Outbreak caused by a single introduction, meaning there were 14 transmission events and 15 total infections. In other words, Rpop=1415=0.933. (B) Outbreak caused by two separate introductions, meaning there were only 13 infection events in the sampling frame, resulting in Rpop=1315=0.867. (TIF) [file pcbi.1009182.s002.tif]

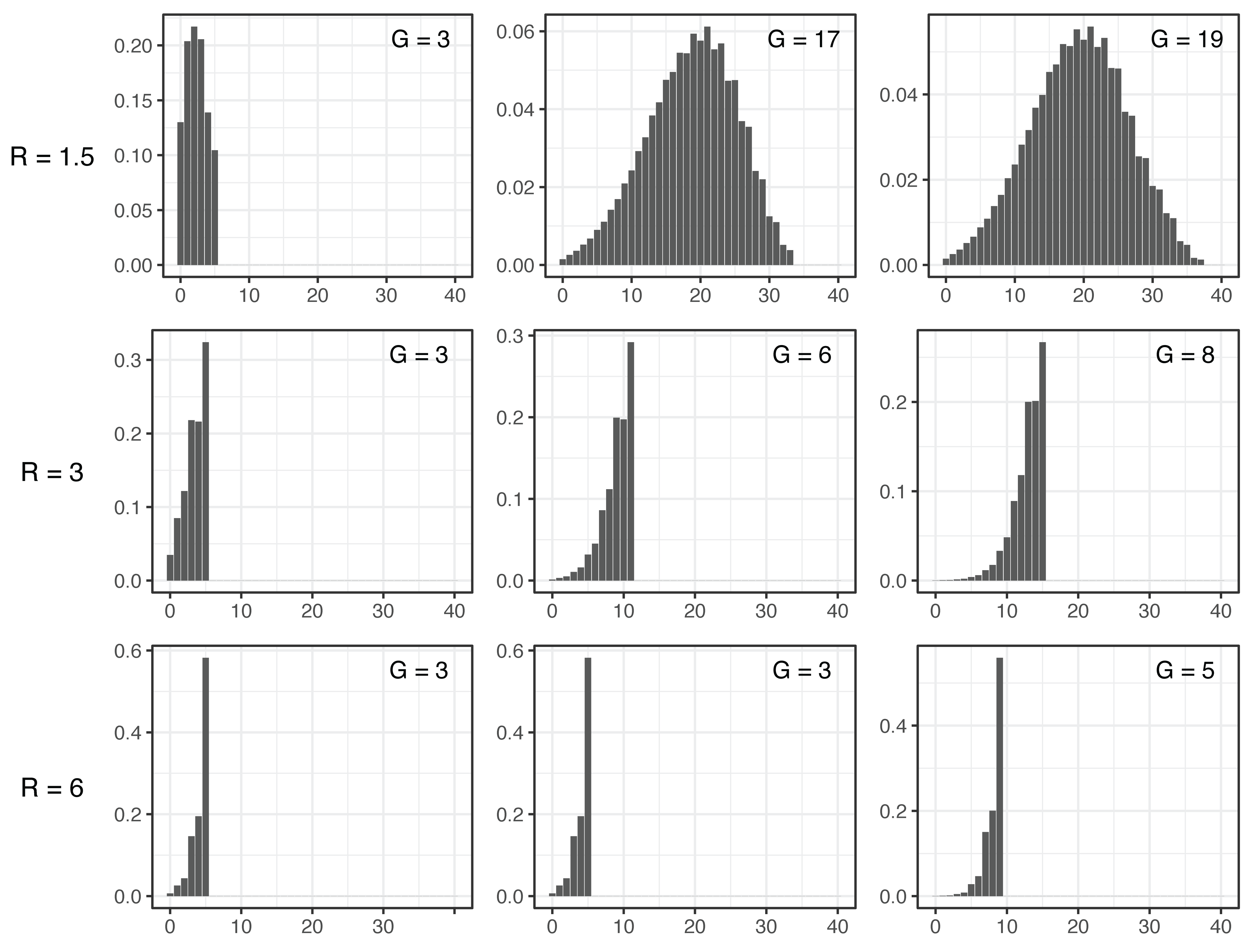

Supplement: S3 Fig — Distribution of the number of generations between infections averaged over 1000 simulated outbreaks with reproduction number R and number of generations of transmission G. Distributions are shown for three values of R (rows). Left column: distribution of generations between infections after 3 generations of transmission; middle column: distribution after ln(1000)/ln(R) generations of transmission (see Methods); right column: distribution after ln(1000)/ln(R)+2 generations of transmission. (TIF) [file pcbi.1009182.s003.tif]

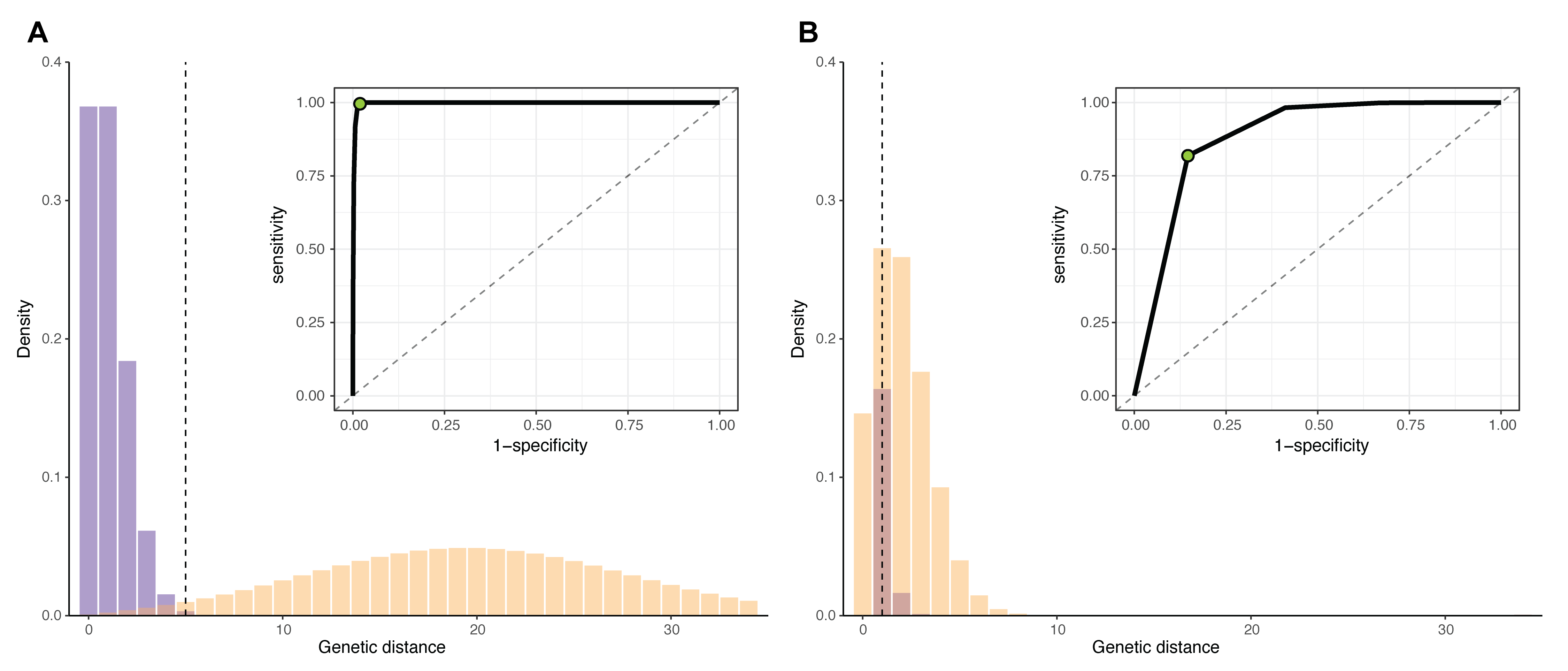

Supplement: S4 Fig — (A) Distribution of genetic distances for linked (purple) and unlinked (yellow) infections for a hypothetical pathogen with substitution rate = 1 substitution/genome/generation and R = 1.5. Inset: receiver operating characteristic (ROC) curve for all possible genetic distance cutoff values. Optimal threshold shown as green dot (ROC) and dashed vertical line (distribution). (B) Distribution of genetic distances for linked and unlinked cases for a hypothetical pathogen with substitution rate = 0.2 mutations/genome/generation and R = 3. Inset: ROC curve for all possible genetic distance cutoff values for this pathogen. The optimal threshold is shown as in (A). (TIF) [file pcbi.1009182.s004.tif]

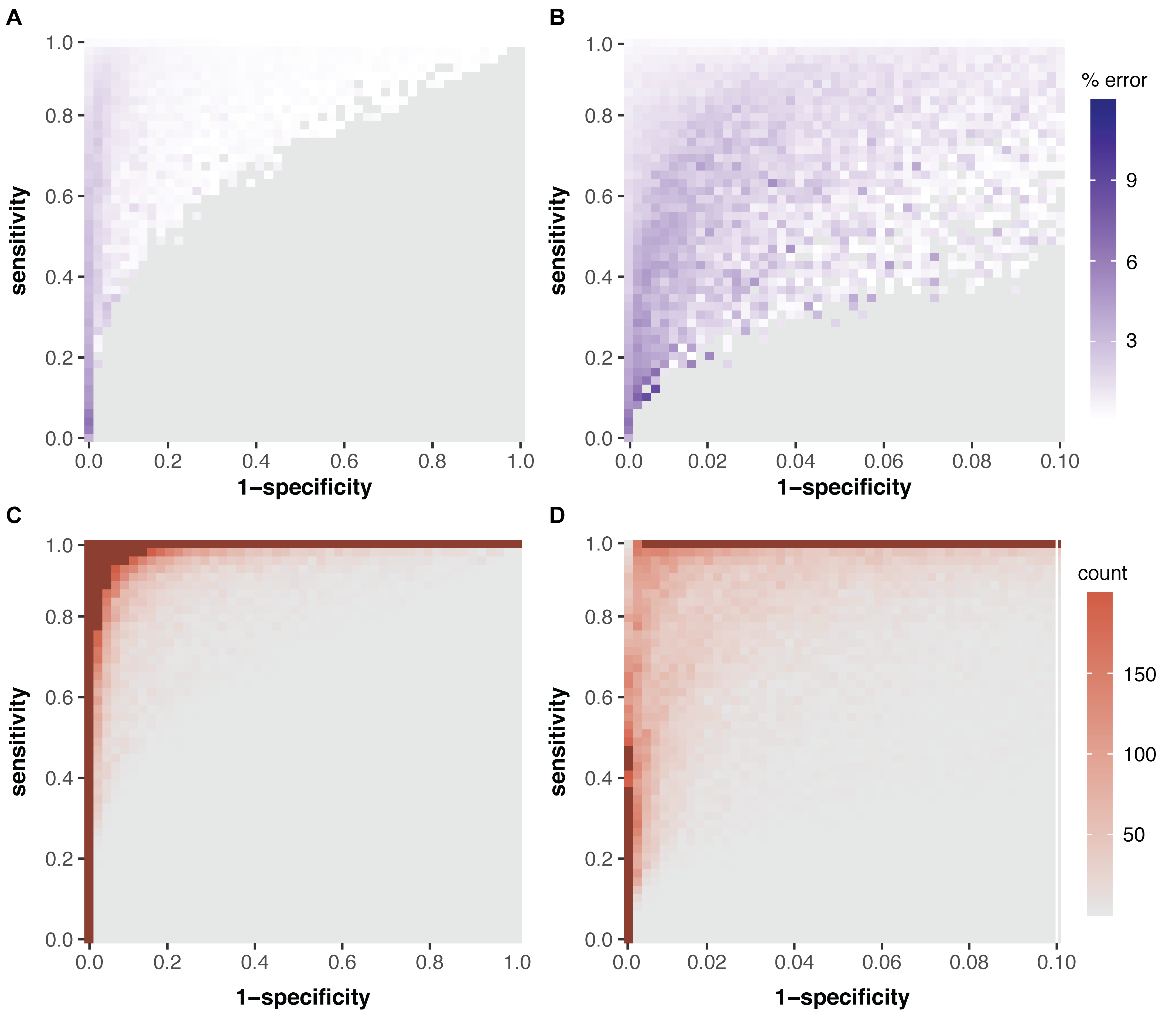

Supplement: S5 Fig — (A) Average false discovery from 10,000 simulated outbreaks (proportion sampled = 0.75) binned by sensitivity and specificity (bin size = 0.02). Grey = no genetic distance thresholds in simulation produced this combination of sensitivity and specificity. (B) Zoom view of (A), with specificity ranging from 0.9–1 (bin size = 0.002). (C) Number of data points with sensitivity and specificity in the desired bins (i.e., number of data points used to calculate average error in panel (A). (D) Zoom view of (C), with specificity ranging from 0.9–1. (TIF) [file pcbi.1009182.s005.tif]

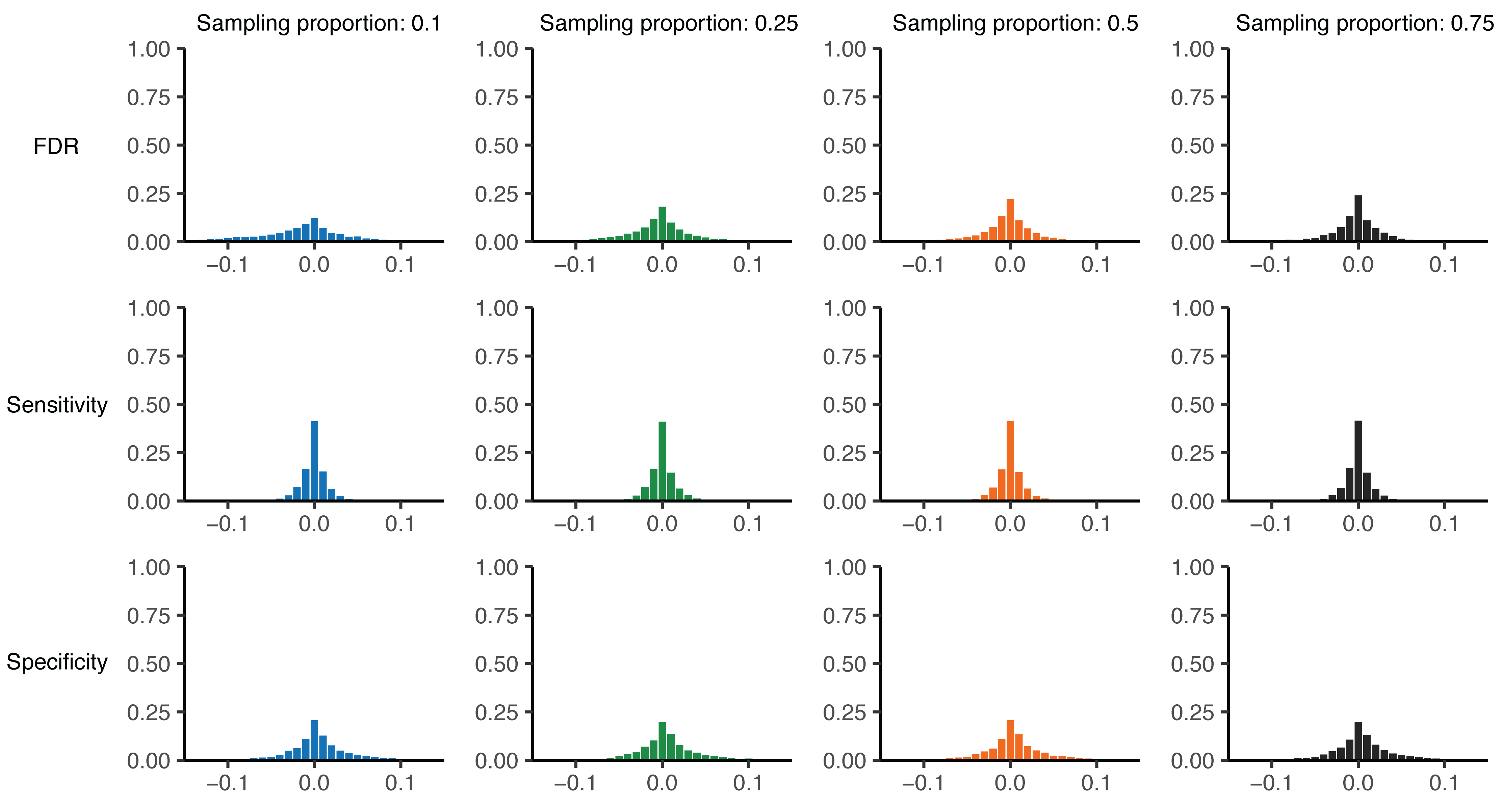

Supplement: S6 Fig — Theoretical minus simulated parameter values for the optimal genetic distance threshold (determined by selecting the threshold for which the point at (1-specificity, sensitivity) is closest to the (0,1) corner) in 10,000 simulations of varying substitution rate and reproductive number for a given sampling proportion. Top row: theoretical minus simulated false discovery rate; middle row: theoretical minus simulated sensitivity; bottom row: theoretical minus simulated specificity. Colors correspond to sampling proportion as in Fig 4. (TIF) [file pcbi.1009182.s006.tif]

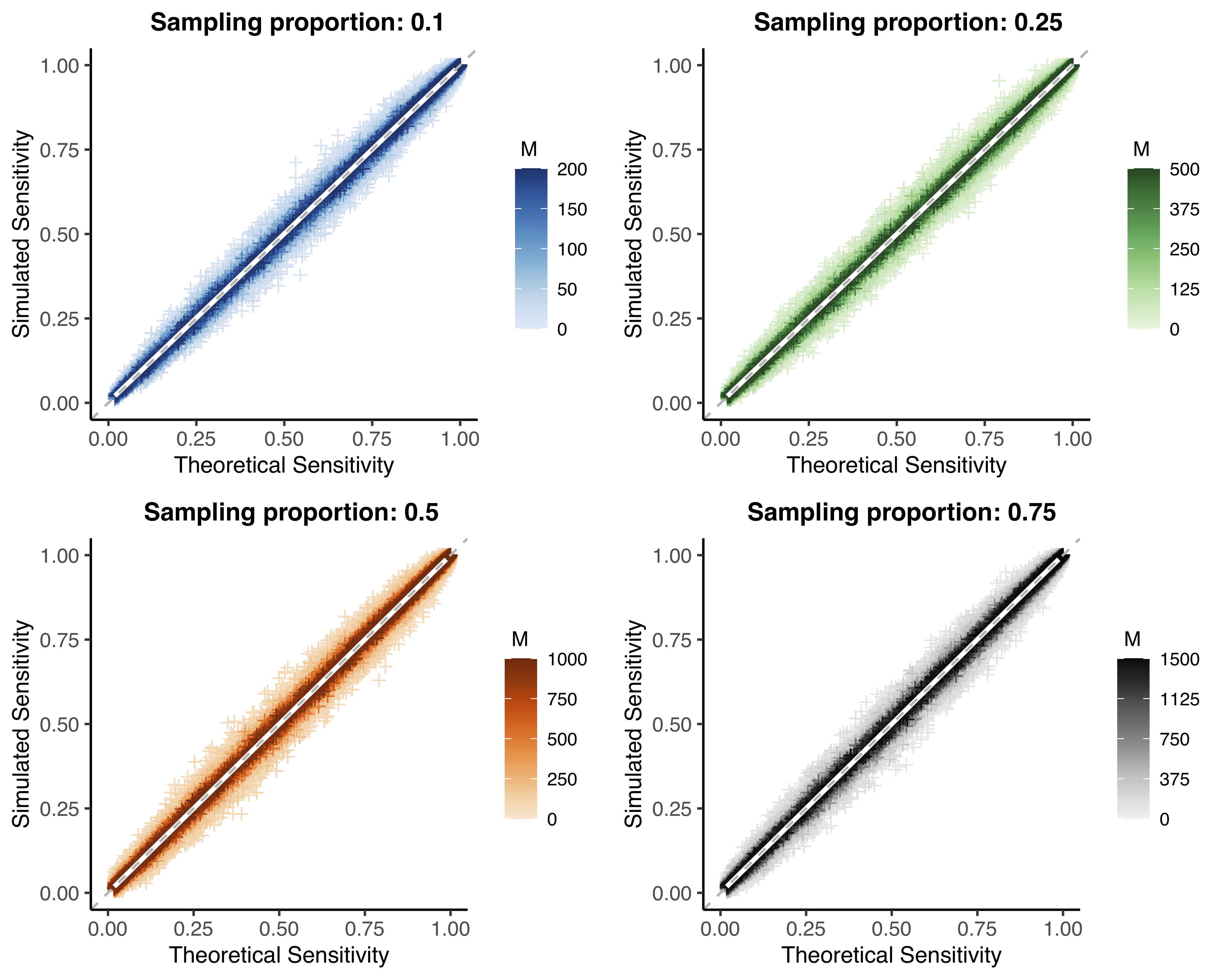

Supplement: S7 Fig — Theoretical versus simulated sensitivity for each genetic distance threshold in 10,000 simulations of varying substitution rate and reproductive number. White line: smoothed conditional mean; grey dashed line: y = x line. Increasing values of the sample size (M) are plotted in darker color. (TIF) [file pcbi.1009182.s007.tif]

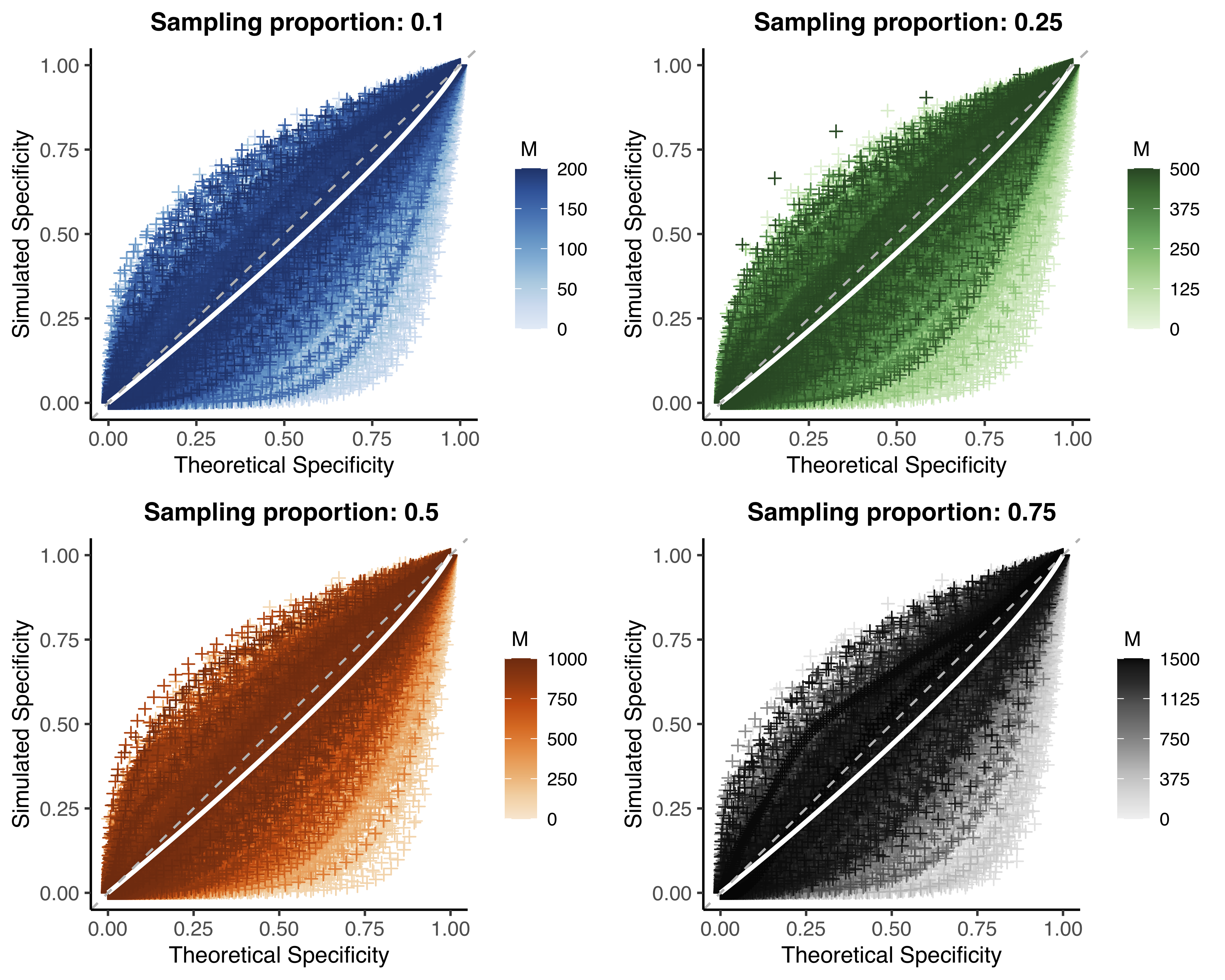

Supplement: S8 Fig — Theoretical versus simulated specificity for each genetic distance threshold in 10,000 simulations of varying substitution rate and reproductive number. Outbreak sizes range from 100–2000, as described in Methods. White line: smoothed conditional mean; grey dashed line: y = x line. Increasing values of the sample size (M) are plotted in darker color. (TIF) [file pcbi.1009182.s008.tif]

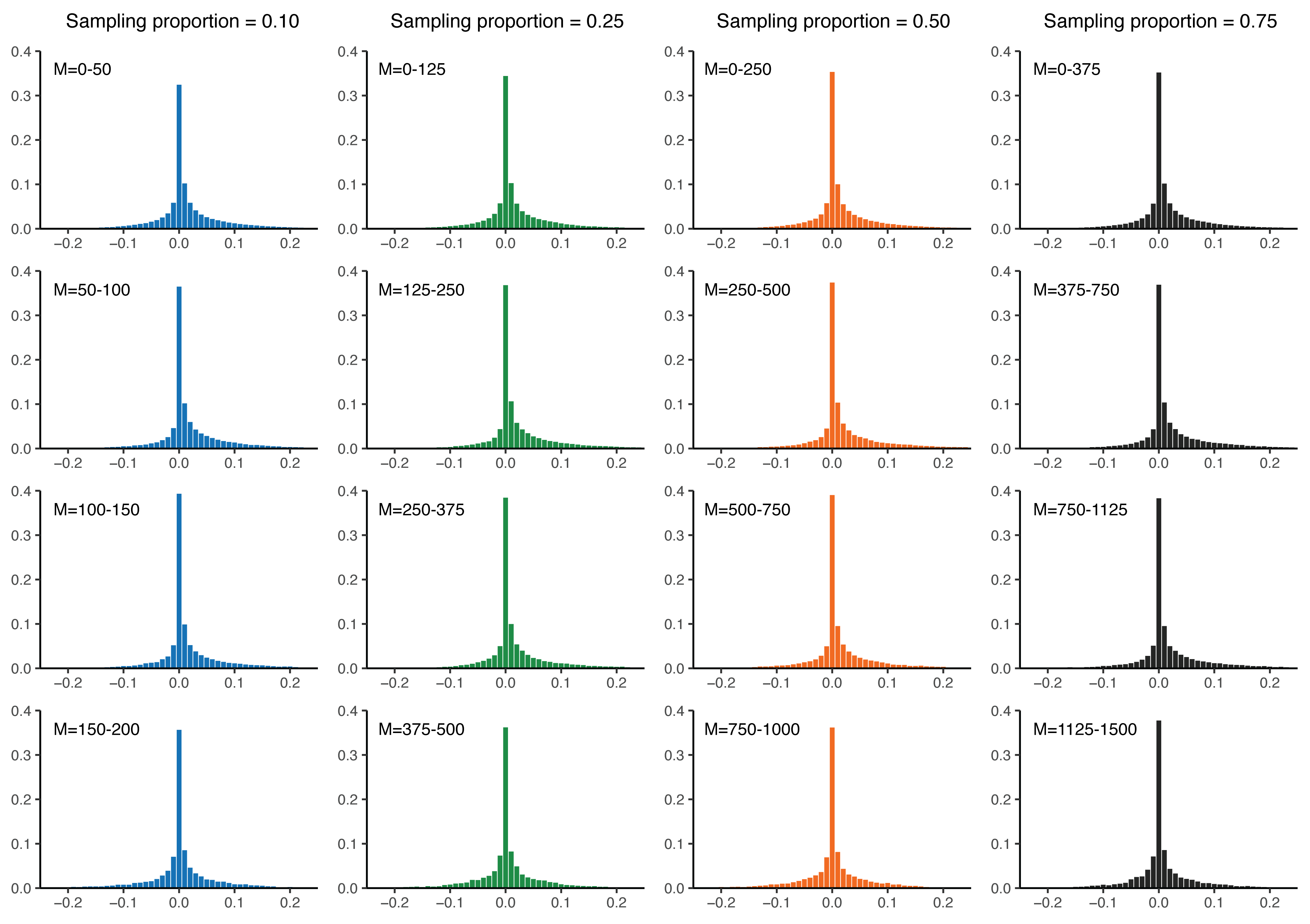

Supplement: S9 Fig — Theoretical minus simulated specificity for each genetic distance threshold in 10,000 simulations of varying substitution rate and reproductive number for a given sampling proportion. Each column represents 10,000 simulations with a specific sampling proportion (colors as in Fig 4) and sample size within each proportion (determined by the final outbreak size) goes from low (top row) to high (bottom row). (TIF) [file pcbi.1009182.s009.tif]

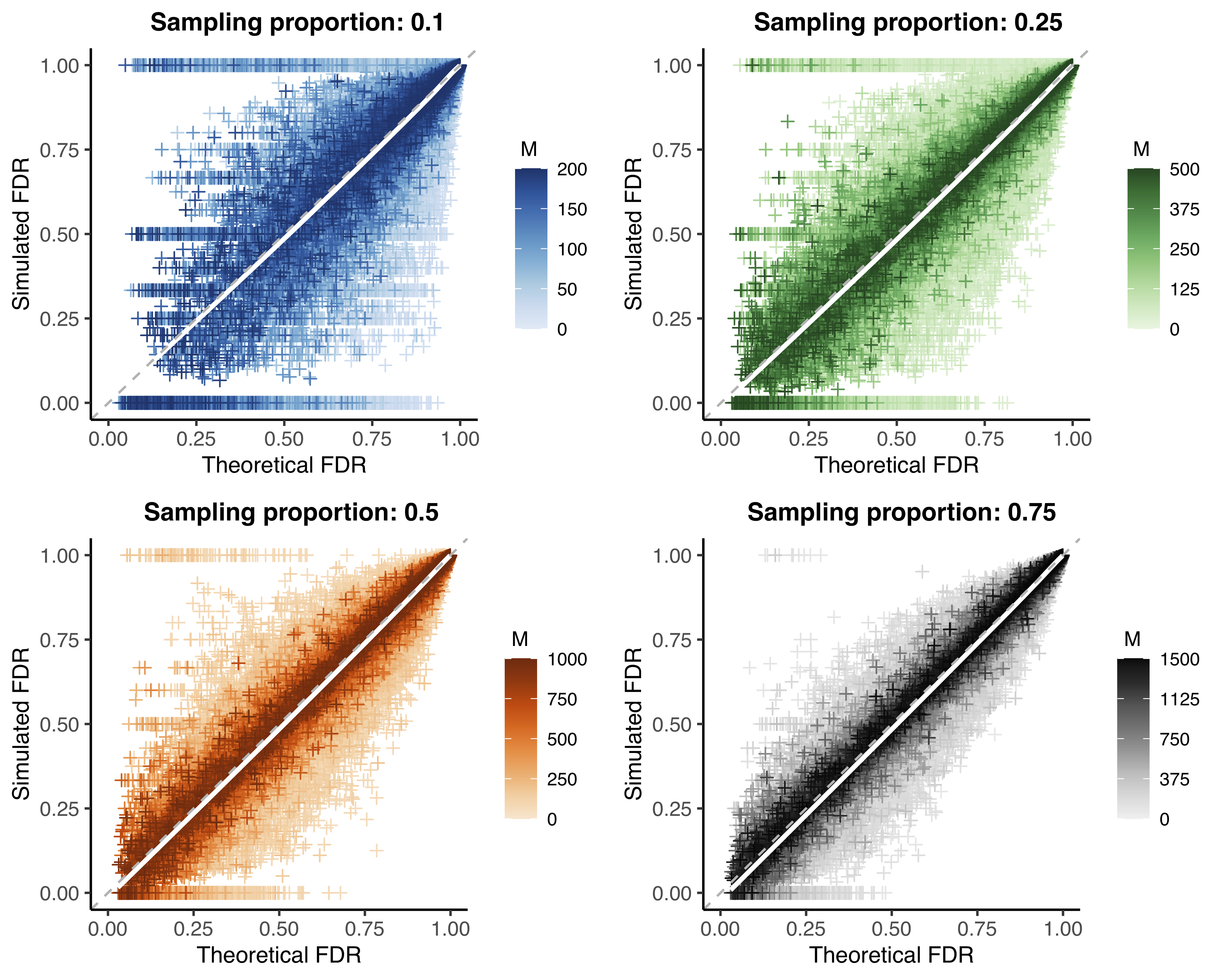

Supplement: S10 Fig — Theoretical versus simulated false discovery rate (FDR) for each genetic distance threshold in 10,000 simulations of varying substitution rate and reproductive number. Theoretical FDR is calculated using the actual distribution of generations between infections from the corresponding simulated outbreak. White line: smoothed conditional mean; grey dashed line: y = x line. Increasing values of the sample size (M) are plotted in darker color. (TIF) [file pcbi.1009182.s010.tif]
