# Supplementary material for: Sample size calculation for phylogenetic case linkage
Source: PLoS Comput Biol. 2021 Jul 6;17(7):e1009182. doi: 10.1371/journal.pcbi.1009182 (PMC8284614; doi:10.1371/journal.pcbi.1009182)
Supplement: S1 Table — (PDF) [file pcbi.1009182.s011.pdf]

**ρ=0.10**	M=0-50	M=50-100	M=100-150	M=150-200	All sample sizes	N
FDR=0.00-0.25	0.2613	0.2172	0.1755	0.1558	**0.2135**	2,269
FDR=0.25-0.50	0.3557	0.2324	0.1736	0.1209	**0.2751**	6,138
FDR=0.50-0.75	0.2645	0.1362	0.0981	0.0717	**0.2057**	11,975
FDR=0.75-1.00	0.0236	0.0074	0.0048	0.0035	**0.0155**	240,978
All FDR Values	**0.044**	**0.0218**	**0.0149**	**0.0107**	**0.032**	261,360
N	140,845	65,386	35,754	19,375	261,360	

**ρ=0.25**	M=0-125	M=125-250	M=250-375	M=375-500	All sample sizes	N
FDR=0.00-0.25	0.1726	0.1219	0.0934	0.0742	**0.1359**	4,420
FDR=0.25-0.50	0.2089	0.1006	0.0677	0.0521	**0.1583**	8,246
FDR=0.50-0.75	0.1268	0.0551	0.0404	0.0308	**0.0979**	13,013
FDR=0.75-1.00	0.0106	0.0031	0.002	0.0014	**0.0069**	241,560
All FDR Values	**0.026**	**0.0106**	**0.0069**	**0.005**	**0.0181**	267,239
N	145,662	64,720	37,176	19,681	267,239	

**ρ=0.50**	M=0-250	M=250-500	M=500-750	M=750-1000	All sample sizes	N
FDR=0.00-0.25	0.1049	0.06	0.0399	0.0314	**0.0799**	5,515
FDR=0.25-0.50	0.1046	0.0442	0.0322	0.0236	**0.079**	8,605
FDR=0.50-0.75	0.0616	0.0279	0.02	0.0149	**0.0478**	13,016
FDR=0.75-1.00	0.0054	0.0017	0.001	0.0007	**0.0035**	241,764
All FDR Values	**0.0141**	**0.0054**	**0.0033**	**0.0022**	**0.0097**	268,900
N	148,403	64,381	35,787	20,329	268,900	

**ρ=0.75**	M=0-375	M=375-750	M=750-1125	M=1125-1500	All sample sizes	N
FDR=0.00-0.25	0.0506	0.0305	0.0213	0.018	**0.0401**	5,696
FDR=0.25-0.50	0.0541	0.0246	0.0163	0.0137	**0.0416**	8,644
FDR=0.50-0.75	0.0331	0.0147	0.0103	0.0085	**0.0259**	13,065
FDR=0.75-1.00	0.003	0.0009	0.0005	0.0004	**0.002**	240,823
All FDR Values	**0.0075**	**0.0028**	**0.0017**	**0.0013**	**0.0052**	268,228
N	150,672	64,274	35,407	17,875	268,228	
