# Supplementary material for: Sample size calculation for phylogenetic case linkage
Source: PLoS Comput Biol. 2021 Jul 6;17(7):e1009182. doi: 10.1371/journal.pcbi.1009182 (PMC8284614; doi:10.1371/journal.pcbi.1009182)
Supplement: S2 Table — (PDF) [file pcbi.1009182.s012.pdf]

**Bias**	ρ=0.10	ρ=0.25	ρ=0.50	ρ=0.75	All ρ values	N
FDR=0.00-0.25	-0.1105	-0.0859	-0.0733	-0.0739	**-0.0823**	23,704
FDR=0.25-0.50	-0.0926	-0.0683	-0.0631	-0.0674	**-0.0715**	35,511
FDR=0.50-0.75	-0.0426	-0.0397	-0.0423	-0.0455	**-0.0425**	54,096
FDR=0.75-1.00	-0.002	-0.002	-0.002	-0.0023	**-0.0021**	952,416
All FDR Values	**-0.008**	**-0.0081**	**-0.0081**	**-0.0088**	**-0.0082**	1,065,727
N	261,360	267,239	268,900	268,228	1,065,727	

**Error**	ρ=0.10	ρ=0.25	ρ=0.50	ρ=0.75	All ρ values	N
FDR=0.00-0.25	0.2551	0.1858	0.1462	0.1298	**0.1678**	23,704
FDR=0.25-0.50	0.2934	0.2059	0.151	0.1343	**0.1891**	35,511
FDR=0.50-0.75	0.2176	0.1395	0.1084	0.1018	**0.1403**	54,096
FDR=0.75-1.00	0.0177	0.0107	0.0084	0.0078	**0.0112**	952,416
All FDR Values	**0.0383**	**0.0279**	**0.0221**	**0.0205**	**0.0271**	1,065,727
N	261,360	267,239	268,900	268,228	1,065,727	
