# Supplementary material for: Sample size calculation for phylogenetic case linkage
Source: PLoS Comput Biol. 2021 Jul 6;17(7):e1009182. doi: 10.1371/journal.pcbi.1009182 (PMC8284614; doi:10.1371/journal.pcbi.1009182)
Supplement: S3 Table — (PDF) [file pcbi.1009182.s013.pdf]

**ρ=0.10**	M=0-50	M=50-100	M=100-150	M=150-200	All sample sizes	N
FDR=0.00-0.25	0.3264	0.2308	0.1735	0.1489	**0.2551**	3,670
FDR=0.25-0.50	0.3627	0.2473	0.1812	0.1542	**0.2934**	7,085
FDR=0.50-0.75	0.2674	0.1597	0.1221	0.1085	**0.2176**	12,728
FDR=0.75-1.00	0.0257	0.01	0.0069	0.0061	**0.0177**	237,877
All FDR Values	**0.0509**	**0.0288**	**0.0186**	**0.0146**	**0.0383**	261,360
N	140,845	65,386	35,754	19,375	261,360	

**ρ=0.25**	M=0-125	M=125-250	M=250-375	M=375-500	All sample sizes	N
FDR=0.00-0.25	0.2356	0.1566	0.1079	0.0941	**0.1858**	5,859
FDR=0.25-0.50	0.2509	0.1552	0.1127	0.0958	**0.2059**	9,231
FDR=0.50-0.75	0.1619	0.1134	0.0846	0.0844	**0.1395**	13,727
FDR=0.75-1.00	0.0144	0.0076	0.0052	0.0053	**0.0107**	238,422
All FDR Values	**0.0367**	**0.0216**	**0.0134**	**0.0115**	**0.0279**	267,239
N	145,662	64,720	37,176	19,681	267,239	

**ρ=0.50**	M=0-250	M=250-500	M=500-750	M=750-1000	All sample sizes	N
FDR=0.00-0.25	0.1858	0.1076	0.0783	0.0647	**0.1462**	6,901
FDR=0.25-0.50	0.1756	0.1207	0.094	0.0849	**0.151**	9,522
FDR=0.50-0.75	0.1194	0.096	0.0793	0.0799	**0.1084**	13,831
FDR=0.75-1.00	0.0106	0.0066	0.0051	0.0048	**0.0084**	238,646
All FDR Values	**0.0283**	**0.0175**	**0.0119**	**0.0099**	**0.0221**	268,900
N	148,403	64,381	35,787	20,329	268,900	

**ρ=0.75**	M=0-375	M=375-750	M=750-1125	M=1125-1500	All sample sizes	N
FDR=0.00-0.25	0.1624	0.0966	0.0645	0.0472	**0.1298**	7,274
FDR=0.25-0.50	0.1517	0.1158	0.0875	0.0762	**0.1343**	9,673
FDR=0.50-0.75	0.1095	0.0957	0.0759	0.0752	**0.1018**	13,810
FDR=0.75-1.00	0.0094	0.0066	0.005	0.0046	**0.0078**	237,471
All FDR Values	**0.0255**	**0.0169**	**0.0113**	**0.0092**	**0.0205**	268,228
N	150,672	64,274	35,407	17,875	268,228	
