# Supplementary material for: Sample size calculation for phylogenetic case linkage
Source: PLoS Comput Biol. 2021 Jul 6;17(7):e1009182. doi: 10.1371/journal.pcbi.1009182 (PMC8284614; doi:10.1371/journal.pcbi.1009182)
Supplement: S4 Table — (PDF) [file pcbi.1009182.s014.pdf]

**Bias**	ρ=0.10	ρ=0.25	ρ=0.50	ρ=0.75	All ρ values	N
FDR=0.00-0.25	0.0062	0.0146	0.013	0.0211	**0.0151**	15,868
FDR=0.25-0.50	0.009	0.0135	0.0167	0.0167	**0.0144**	31,770
FDR=0.50-0.75	0.0153	0.0157	0.0125	0.0132	**0.0141**	50,302
FDR=0.75-1.00	0.0011	0.0011	0.001	0.0011	**0.0011**	967,787
All FDR Values	**0.002**	**0.0024**	**0.0023**	**0.0025**	**0.0023**	1,065,727
N	261,360	267,239	268,900	268,228	1,065,727	

**Error**	ρ=0.10	ρ=0.25	ρ=0.50	ρ=0.75	All ρ values	N
FDR=0.00-0.25	0.2192	0.1494	0.1071	0.0792	**0.1233**	15,868
FDR=0.25-0.50	0.2766	0.1711	0.1006	0.075	**0.1448**	31,770
FDR=0.50-0.75	0.2131	0.1121	0.0694	0.0569	**0.1104**	50,302
FDR=0.75-1.00	0.0168	0.0086	0.0059	0.005	**0.0091**	967,787
All FDR Values	**0.0331**	**0.0207**	**0.0138**	**0.0112**	**0.0196**	1,065,727
N	261,360	267,239	268,900	268,228	1,065,727	
