# Supplementary material for: Sample size calculation for phylogenetic case linkage
Source: PLoS Comput Biol. 2021 Jul 6;17(7):e1009182. doi: 10.1371/journal.pcbi.1009182 (PMC8284614; doi:10.1371/journal.pcbi.1009182)
Supplement: S1 Text — (PDF) [file pcbi.1009182.s015.pdf]

Text S1

Deriving probability of transmission given linkage

We begin by defining two matrices containing binary response variables. The first matrix $\mathbf{Y}$ contains the variable y_{ij}, which indicates that infection i and infection j are linked through a direct transmission event (i.e., i infected j or vice versa). Matrix $\mathbf{Y}$ has dimensions $N \times N$, where N is the population (i.e., final outbreak) size. The second matrix $\mathbf{Z}$ contains the variable z_{ij}, which indicates inferred linkage between infections i and j based on some phylogenetic criteria. Matrix $\mathbf{Z}$ has dimensions $M \times M$, where M is the sample size and $M \subset N$.

Our aim is to determine the quantity $\Pr(y_{ij} \mid z_{ij})$, which is the probability that infection i is linked by transmission to infection j (making i and j a true transmission pair), given that they have been linked by some phylogenetic criteria. We start by making a number of assumptions that simplify the derivation, and we relax each of these assumptions in turn.

A Single link, single true transmission, and perfect sensitivity

A.1 Assumptions

We make the following simplifying assumptions:

1. Each infection i is linked by transmission to only one other infection j in the population (N).
2. Each infection i is linked by the linkage criteria to only one other infection j in the sampled population (M).
3. The sensitivity of the linkage criteria is equal to 1 when both the infector and infectee have been sampled. If infection i is truly linked by transmission to infection j and both infections are found in sample M, then $y_{ij} = 1$ by definition. Under this assumption of perfect sensitivity, $z_{ij} = 1$ as well.

A.2 Derivation of the probability of transmission given linkage

Under the assumptions above, we can show that:

$$\Pr(y_{ij} \mid z_{ij}) = \frac{\Pr(y_{ij}, z_{ij})}{\Pr(z_{ij})} = \frac{\Pr(y_{ij}, z_{ij})}{\Pr(y_{ij}, z_{ij}) + \Pr(\neg y_{ij}, z_{ij})}$$

However, we must also account for the uncertainty of sampling the true transmission partner of i (the infection directly linked to i by transmission, i.e., either its infector or infectee). We define define S_i as the probability that the true transmission partner of i has been sampled from the population N and apply the law of total probability accordingly:

$$= \frac{\Pr(\cancelto{1}{y_{ij}, z_{ij} \mid S_i})\Pr(S_i) + \Pr(\cancelto{0}{y_{ij}, z_{ij} \mid \neg S_i})\Pr(\neg S_i)}{\left[\begin{array}{l}\Pr(\cancelto{1}{y_{ij}, z_{ij} \mid S_i})\Pr(S_i) + \Pr(\cancelto{0}{y_{ij}, z_{ij} \mid \neg S_i})\Pr(\neg S_i) + \\ \Pr(\cancelto{0}{\neg y_{ij}, z_{ij} \mid S_i})\Pr(S_i) + \Pr(\neg y_{ij}, z_{ij} \mid \neg S_i)\Pr(\neg S_i)\end{array}\right]}$$

$$= \frac{\Pr(S_i)}{\Pr(S_i) + \Pr(\neg y_{ij}, z_{ij} \mid \neg S_i)\Pr(\neg S_i)}$$

We know that $\Pr(S_i)$ is equal to the sampling fraction ($\frac{M}{N}$), which we define as ρ:

$$= \frac{\rho}{\rho + \Pr(\neg y_{ij}, z_{ij} \mid \neg S_i)(1-\rho)}$$

The term $\Pr(\neg y_{ij}, z_{ij} \mid \neg S_i)$ is the probability that i is linked to j when infection i is not the true transmission partner of j, given that the true partner of i is not in the sample M. Given our assumption that each infection is linked to exactly one other infection by the phylogenetic criteria, the probability of this (incorrect) link between infections i and j is equal the probability that the remaining $M-1$ other possible (incorrect) links do not occur, which can be written as $(1-\chi^{M-1})$, where χ is the specificity of the linkage criteria:

$$= \frac{\rho}{\rho + (1-\chi^{M-1})(1-\rho)}$$

Therefore, the probability of transmission given linkage assuming perfect sensitivity, single transmission, and single linkage is:

$$\Pr(y_{ij} \mid z_{ij}) = \frac{\rho}{\rho + (1-\chi^{M-1})(1-\rho)} \tag{1}$$

The probability spaces in Equation 1 above can also be represented by the conceptual diagram below:

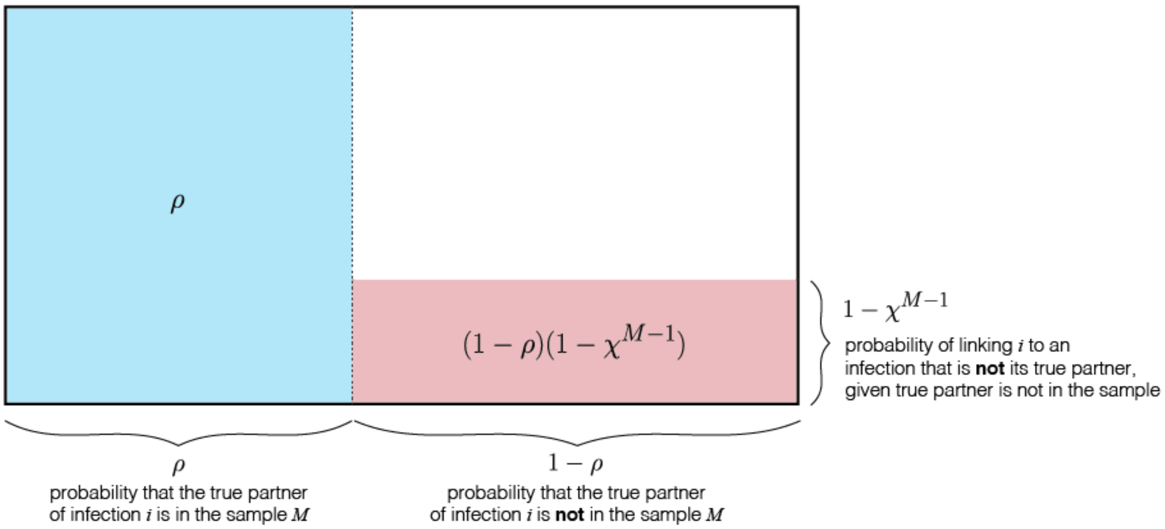

A.3 Calculating the expected number of pairs in the sample

Given the number of pairs identified from the linkage criteria, the expected number of those pairs that represent true transmission pairs is:

$$\mathbb{E}[\text{number of true pairs}] = \mathbb{E}[\text{Number of pairs observed}] \times \Pr(\text{an observed pair is true}).$$

We have defined ρ as the probability of selecting any individual from the population N. Therefore, if we assume a large population size, the probability of sampling both infection i and its true transmission partner is equal to ρ^2. Under

our first assumption—that i is linked by transmission to only one other infection j—the total number of pairs in the population is equal to $\frac{N}{2}$. We also know that $\rho = \frac{M}{N}$, so the total number of true transmission pairs in the sample is:

$$\mathbb{E}[\text{number of true pairs}] = \rho^2 \times \frac{N}{2} = \rho^2 \times \frac{1}{2}\frac{M}{\rho} = \frac{M}{2}\rho \tag{2}$$

Rearranging and substituting Equation 1 for $\Pr(\text{an observed pair is true})$:

$$\begin{aligned}
\mathbb{E}[\text{number of pairs observed}] &= \frac{\mathbb{E}[\text{number of true pairs}]}{\Pr(\text{an observed pair is true})} \\
&= \frac{\frac{M}{2}\rho}{\rho/[\rho + (1-\chi^{M-1})(1-\rho)]} \\
&= \frac{M}{2}[\rho + (1-\chi^{M-1})(1-\rho)]
\end{aligned}$$

Therefore, the expected number of pairs observed assuming perfect sensitivity, single linkage, and single transmission is:

$$\mathbb{E}[\text{number of pairs observed}] = \frac{M}{2}[\rho + (1-\chi^{M-1})(1-\rho)] \tag{3}$$

Under our simplifying assumptions, Equation 3 reveals two important principles:

1. The quantity $\mathbb{E}[\text{number of pairs observed}]$ increases more rapidly than $\mathbb{E}[\text{number of true pairs}]$ as M increases. Therefore, the false discovery rate increases as M increases, all else being equal.
2. Both $\mathbb{E}[\text{number of pairs observed}]$ and $\Pr(y_{ij} \mid z_{ij})$ are highly dependent on the value of χ, the specificity of the linkage criteria.

B Single link and single true transmission

B.1 Assumptions

In this section, we preserve the first two assumptions from the prior section and relax our assumption of perfect sensitivity. Our remaining assumptions are:

1. Each infection i is linked by transmission to only one other infection j in the population (N).
2. Each infection i is linked by the linkage criteria to only one other infection j in the sampled population (M).

B.2 Derivation of the probability of transmission given linkage

When perfect sensitivity is relaxed, we must account for both the uncertainty that the true transmission partner of i is in sample M and the uncertainty that we correctly identify this pairing when both infections are sampled. Thus, we rewrite Equation 1 with additional terms to account for the increased number of potential outcomes.

$$\Pr(y_{ij} \mid z_{ij}) = \frac{\Pr(y_{ij}, z_{ij})}{\Pr(z_{ij})} = \frac{\Pr(y_{ij}, z_{ij})}{\Pr(y_{ij}, z_{ij}) + \Pr(\neg y_{ij}, z_{ij})}$$

$$= \frac{\Pr(y_{ij}, z_{ij} \mid S_i)\Pr(S_i) + \Pr(\cancelto{0}{y_{ij}, z_{ij} \mid \neg S_i})\Pr(\neg S_i)}{\begin{bmatrix} \Pr(y_{ij}, z_{ij} \mid S_i)\Pr(S_i) + \Pr(\cancelto{0}{y_{ij}, z_{ij} \mid \neg S_i})\Pr(\neg S_i) + \\ \Pr(\neg y_{ij}, z_{ij} \mid S_i)\Pr(S_i) + \Pr(\neg y_{ij}, z_{ij} \mid \neg S_i)\Pr(\neg S_i) \end{bmatrix}}$$

$$= \frac{\Pr(y_{ij}, z_{ij} \mid S_i)\Pr(S_i)}{\Pr(y_{ij}, z_{ij} \mid S_i)\Pr(S_i) + \Pr(\neg y_{ij}, z_{ij} \mid S_i)\Pr(S_i) + \Pr(\neg y_{ij}, z_{ij} \mid \neg S_i)\Pr(\neg S_i)}$$

The specificity of the linkage criteria (defined here as η) is the probability that a link is correctly identified between infection i and its true transmission partner when both are in the sample, or $\Pr(y_{ij}, z_{ij} \mid S_i)$. Substituting this and the previously-defined $\Pr(S_i) = \rho$, we get:

$$= \frac{\eta\rho}{\eta\rho + \Pr(\neg y_{ij}, z_{ij} \mid S_i)\rho + \Pr(\neg y_{ij}, z_{ij} \mid \neg S_i)(1-\rho).}$$

We know that $\Pr(\neg y_{ij}, z_{ij} \mid S_i)$ is the probability that the true partner of infection i is in the sample M, but that i is incorrectly linked to j, an infection that is not its true transmission partner. This is expressed as the probability of i not being linked to its true (sampled) transmission partner $(1-\eta)$ or any of the $M-2$ other sampled infections $(1-\chi^{M-2})$. In this derivation, we again assume that each infection is linked to exactly one other, so avoiding linkage with all other sampled infections implies that i is linked to the remaining infection j (in this case, not its true transmission partner). If the true partner of i is not in the sample $\left(\Pr(\neg y_{ij}, z_{ij} \mid \neg S_i)\right)$, the probability of linking i to one other sampled infection that is not its true partner is simply $1-\chi^{M-1}$, as previously defined.

Therefore, the probability of transmission given linkage assuming single transmission and single linkage is:

$$\Pr(y_{ij} \mid z_{ij}) = \frac{\eta\rho}{\eta\rho + (1-\chi^{M-2})(1-\eta)\rho + (1-\chi^{M-1})(1-\rho)} \tag{4}$$

The probability spaces in Equation 4 above can also be represented by the conceptual diagram below:

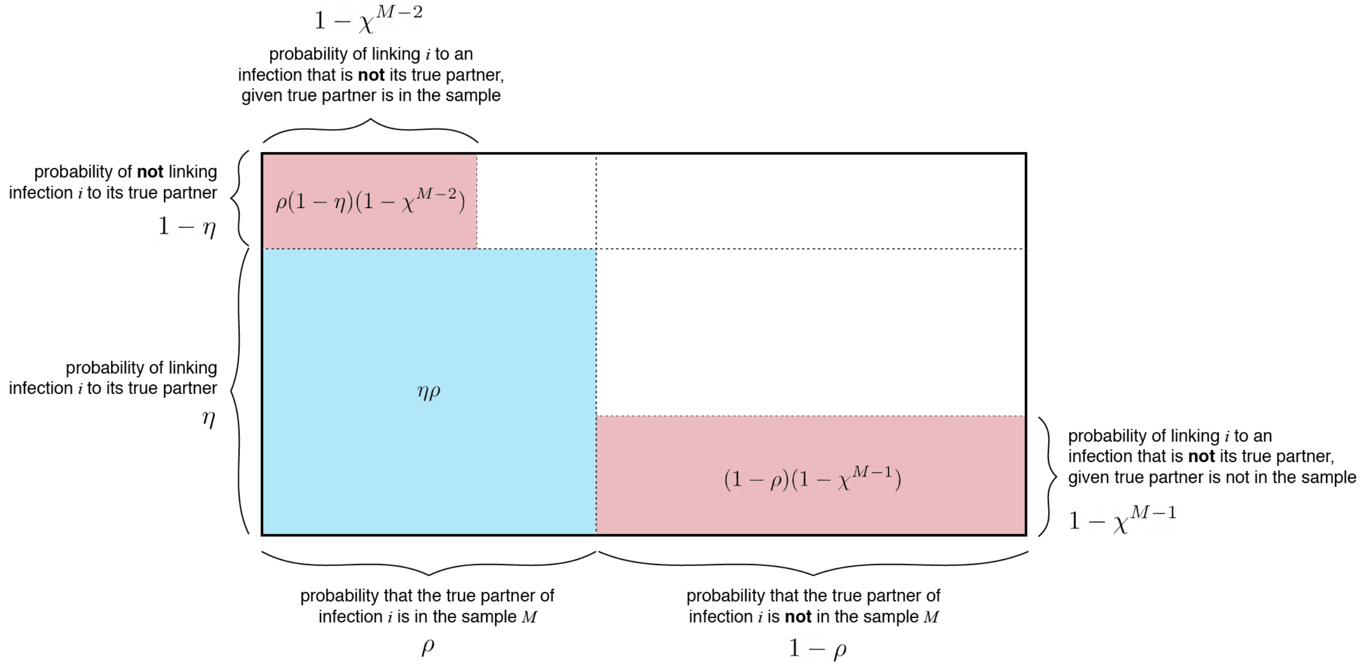

This derivation makes the implicit assumption that sensitivity is independent of both sample size and specificity ($M \perp \eta \perp \chi$). This is unlikely to be true in a real transmission scenario where infection i is closely related to multiple other infections, but allows us to approximate the probability that an identified transmission link is true given our other assumptions.

B.3 Calculating the expected number of pairs in the sample

We now re-write Equation 2 with the sensitivity assumption relaxed:

$$\mathbb{E}[\text{number of true pairs}] = \eta\rho^2 \times \frac{N}{2} = \eta\rho^2 \times \frac{1}{2}\frac{M}{\rho} = \frac{M}{2}\eta\rho \tag{5}$$

Where the probability that an infection and its transmission partner are both in the sample is still ρ^2, but we must now also include the probability of that pair being correctly identified by the linkage criteria, η. We can again calculate the expected number of pairs observed, this time incorporating the sensitivity:

$$\mathbb{E}[\text{number of pairs observed}] = \frac{\mathbb{E}[\text{number of true pairs}]}{\Pr(\text{an observed pair is true})}$$

$$= \frac{\frac{M}{2}\eta\rho}{\eta\rho/[\eta\rho + \rho(1-\eta)(1-\chi^{M-2}) + (1-\rho)(1-\chi^{M-1})]}$$

$$= \frac{M}{2}[\eta\rho + \rho(1-\eta)(1-\chi^{M-2}) + (1-\rho)(1-\chi^{M-1})]$$

Therefore, the expected number of pairs observed, assuming single linkage and single transmission is:

$$\mathbb{E}[\text{number of pairs observed}] = \frac{M}{2}[\eta\rho + \rho(1-\eta)(1-\chi^{M-2}) + (1-\rho)(1-\chi^{M-1})] \quad (6)$$

As before (see Equation 3), when all other parameters are held constant, the false discovery rate will increase as the sample size M increases. This is because the number of observed pairs increases more rapidly than the number of true pairs. Further, with imperfect sensitivity, increasing the sample size M has an even more substantial effect (due to an additional term containing 1-χ^M) on the expected number of pairs observed than before, thus more quickly increasing the false discovery rate.

C Single link and multiple true transmissions

C.1 Assumptions

Thus far, we have assumed that every infection i has been connected by transmission to exactly one other infection; in other words, that i is either an infector or infectee, but not both. However, we are often interested in capturing all transmission partners of i, including its infector and all infectees. Therefore, we relax the single transmission assumption and calculate the probability of correctly identifying a true pair given that i has transmitted to R (the pathogen reproductive number) other individuals in the population. However, we maintain that each individual has been infected by exactly one other individual, i.e., that multiple infections are not possible. Therefore, each infection i has on average $R+1$ true transmission partners, and we define k as the number of these true partners that are in the sample M.

As a result, we remain with just one of our original assumptions:

1. Each infection i is linked by the linkage criteria to only one other infection j in the sampled population (M).

C.2 Derivation of the probability of transmission given linkage

Derivation for a given value of k

If there are k individuals in sample M that are true transmission partners of infection i, then the probability an identified link is true given that any infection i has k sampled transmission partners is:

$$
\begin{aligned}
\Pr(y_{ij} \mid z_{ij}, k) &= \frac{\Pr(y_{ij}, z_{ij}, k)}{\Pr(z_{ij}, k)} \\
&= \frac{\Pr(y_{ij}, z_{ij}, k)}{\Pr(y_{ij}, z_{ij}, k) + \Pr(\neg y_{ij}, z_{ij}, k)} \\
&= \frac{\Pr(y_{ij}, z_{ij} \mid k)\Pr(k)}{\Pr(y_{ij}, z_{ij} \mid k)\Pr(k) + \Pr(\neg y_{ij}, z_{ij} \mid k)\Pr(k)} \\
&= \frac{\Pr(y_{ij}, z_{ij} \mid k)}{\Pr(y_{ij}, z_{ij} \mid k) + \Pr(\neg y_{ij}, z_{ij} \mid k)}
\end{aligned}
$$

We can show that the probability that infection i is not linked (by the linkage citeria) to any of its k true partners is $(1-\eta)^k$, so the probability that infection i is linked to at least one of its k true partners in the sample is $1-(1-\eta)^k$. Because we still assume that the linkage criteria will identify exactly one link for each infection i, this is equivalent to the probability $\Pr(y_{ij}, z_{ij} \mid k)$:

$$
= \frac{[1-(1-\eta)^k]}{[1-(1-\eta)^k] + \Pr(\neg y_{ij}, z_{ij} \mid k)}
$$

Similarly, the probability that infection i is incorrectly linked to another infection is the probability it is not linked to any of its true partners ($(1-\eta)^k$) times the probability of not linking to any of the other sampled infections ($1-\chi^{M-1-k}$).

Therefore, the probability of transmission given linkage, assuming k sampled partners and single linkage is:

$$\Pr(y_{ij} \mid z_{ij}, k) = \frac{[1-(1-\eta)^k]}{[1-(1-\eta)^k] + (1-\eta)^k(1-\chi^{M-1-k})} \tag{7}$$

Derivation for all possible values of k

We can extend Equation 7 to include all possible values of k for a given infection i:

$$\begin{aligned}
\Pr(y_{ij} \mid z_{ij}) &= \sum_{k=0}^{\infty} \Pr(y_{ij} \mid z_{ij}, k) \Pr(k \mid z_{ij}) \\
&= \sum_{k=0}^{\infty} \Pr(y_{ij} \mid z_{ij}, k) \frac{\Pr(z_{ij} \mid k) \Pr(k)}{\Pr(z_{ij})} \\
&= \frac{1}{\Pr(z_{ij})} \sum_{k=0}^{\infty} \Pr(y_{ij} \mid z_{ij}, k) \Pr(z_{ij} \mid k) \Pr(k) \\
&= \frac{1}{\Pr(z_{ij})} \sum_{k=0}^{\infty} \Pr(y_{ij}, z_{ij} \mid k) \Pr(k) \\
&= \frac{1}{\sum_{k=0}^{\infty} \Pr(z_{ij} \mid k) \Pr(k)} \sum_{k=0}^{\infty} \Pr(y_{ij}, z_{ij} \mid k) \Pr(k) \\
&= \frac{1}{\sum_{k=0}^{\infty} [\Pr(y_{ij}, z_{ij} \mid k) + \Pr(\neg y_{ij}, z_{ij} \mid k)] \Pr(k)} \sum_{k=0}^{\infty} \Pr(y_{ij}, z_{ij} \mid k) \Pr(k) \\
&= \frac{\sum_{k=0}^{\infty} \Pr(k) \Pr(y_{ij}, z_{ij} \mid k)}{\sum_{k=0}^{\infty} \Pr(k) [\Pr(y_{ij}, z_{ij} \mid k) + \Pr(\neg y_{ij}, z_{ij} \mid k)]} \\
&= \frac{\sum_{k=0}^{\infty} \Pr(k)(1-(1-\eta)^k)}{\sum_{k=0}^{\infty} \Pr(k)[(1-(1-\eta)^k) + (1-\eta)^k(1-\chi^{M-1-k})]}
\end{aligned} \tag{8}$$

As a check on the formulation of Equation 8, let there be only one true transmission partner for infection i. In this instance, $k = 1$ occurs with probability ρ (the probability that this single partner is in the sample) and $k = 0$ occurs with probability $1 - \rho$:

$$
\begin{aligned}
\Pr(y_{ij} \mid z_{ij}) &= \frac{\sum_{k=0}^{1} \Pr(k)(1-(1-\eta)^k)}{\sum_{k=0}^{1} \Pr(k)[(1-(1-\eta)^k) + (1-\eta)^k(1-\chi^{M-1-k})]} \\
&= \frac{[(1-\rho)(1-\cancelto{0}{(1-\eta)^0}) + \rho(1-(1-\eta)^1)]}{\begin{bmatrix} (1-\rho)[(1-\cancelto{0}{(1-\eta)^0}) + \cancelto{1}{(1-\eta)^0}(1-\chi^{M-1-0})] + \\ \rho[(1-(1-\eta)^1) + (1-\eta)^1(1-\chi^{M-1-1})] \end{bmatrix}} \\
&= \frac{\eta\rho}{(1-\rho)(1-\chi^{M-1}) + \eta\rho + \rho(1-\eta)(1-\chi^{M-2})}
\end{aligned} \tag{9}
$$

This result is equivalent to Equation 4 above, which was also derived under the assumption that each infection i is truly connected by transmission to exactly one other infection.

Therefore, we can conclude that the probability of transmission given linkage, assuming single linkage and for all possible values of k transmission links in the sample, is:

$$
\Pr(y_{ij} \mid z_{ij}) = \frac{\sum_{k=0}^{\infty} \Pr(k)(1-(1-\eta)^k)}{\sum_{k=0}^{\infty} \Pr(k)[(1-(1-\eta)^k) + (1-\eta)^k(1-\chi^{M-1-k})]} \tag{10}
$$

Derivation if k is poisson-distributed

In an infectious disease outbreak, it may be difficult or impossible to know the true number of transmission partners in the sample for any given infection. Therefore, we use the population average for the number of secondary infections, which we define here are R_{pop}. Note that we use R_{pop} instead of the traditional R_e because R_e has a specific meaning with regards to disease susceptibility in the population, and here we mean the average number of secondary infections of each infection in a finite population. As discussed in the main text, in practice R_{pop} is always less than one.

We draw k from a Poisson distribution with mean $\lambda = \rho(R_{\text{pop}} + 1)$. Here, $R_{\text{pop}} + 1$ is the total number of transmission links for a given sampled infection i; we add one because infection i is linked to the R_{pop} individuals he/she infects as well as to his/her infector (note that multiple infections are not allowed under the assumption that each infected individual is infected by exactly one individual). We multiply by ρ to account for the probability that each of these true transmission partners is actually included in the sample.

We incorporate the Poisson representation of the number of true transmission links in the sample with the Poisson probability density function:

$$
\Pr(k \mid \lambda) = \lambda^k e^{-\lambda} \frac{1}{k!}. \tag{11}
$$

Returning to the result of the derivation in Equation 8, we now have:

$$\Pr(y_{ij} \mid z_{ij}) =$$

$$= \frac{\sum_{k=0}^{\infty} \Pr(k \mid \lambda)(1-(1-\eta)^k)}{\sum_{k=0}^{\infty} \Pr(k \mid \lambda)[(1-(1-\eta)^k) + (1-\eta)^k(1-\chi^{M-1-k})]}$$

$$= \frac{\sum_{k=0}^{\infty} \lambda^k e^{-\lambda}\frac{1}{k!}(1-(1-\eta)^k)}{\sum_{k=0}^{\infty} \lambda^k e^{-\lambda}\frac{1}{k!}[(1-(1-\eta)^k) + (1-\eta)^k(1-\chi^{M-1-k})]}$$

$$= \frac{\sum_{k=0}^{\infty}\left[\lambda^k e^{-\lambda}\frac{1}{k!}\right] - \sum_{k=0}^{\infty}\left[\lambda^k e^{-\lambda}\frac{1}{k!}(1-\eta)^k\right]}{\sum_{k=0}^{\infty}\left[\lambda^k e^{-\lambda}\frac{1}{k!}\right] - \sum_{k=0}^{\infty}\left[\lambda^k e^{-\lambda}\frac{1}{k!}(1-\eta)^k\right] + \sum_{k=0}^{\infty}\left[\lambda^k e^{-\lambda}\frac{1}{k!}(1-\eta)^k(1-\chi^{M-1-k})\right]}$$

we know that $\left[\lambda^k e^{-\lambda}\frac{1}{k!}\right]$ is the probability density function of a Poisson distribution with mean λ, therefore the sum of this expression over all values of k is, by definition, equal to one.

$$= \frac{1 - \sum_{k=0}^{\infty}\left[\lambda^k e^{-\lambda}\frac{1}{k!}(1-\eta)^k\right]}{1 - \sum_{k=0}^{\infty}\left[\lambda^k e^{-\lambda}\frac{1}{k!}(1-\eta)^k\right] + \sum_{k=0}^{\infty}\left[\lambda^k e^{-\lambda}\frac{1}{k!}(1-\eta)^k(1-\chi^{M-1-k})\right]}$$

$$= \frac{1 - \sum_{k=0}^{\infty}\left[\lambda^k e^{-\lambda}\frac{1}{k!}(1-\eta)^k\right]}{1 - \sum_{k=0}^{\infty}\left[\lambda^k e^{-\lambda}\frac{1}{k!}(1-\eta)^k\right] + \sum_{k=0}^{\infty}\left[\lambda^k e^{-\lambda}\frac{1}{k!}(1-\eta)^k\right] - \sum_{k=0}^{\infty}\left[\lambda^k e^{-\lambda}\frac{1}{k!}(1-\eta)^k(\chi^{M-1-k})\right]}$$

$$= \frac{1 - \sum_{k=0}^{\infty}\left[\lambda^k e^{-\lambda}\frac{1}{k!}(1-\eta)^k\right]}{1 - \sum_{k=0}^{\infty}\left[\lambda^k e^{-\lambda}\frac{1}{k!}(1-\eta)^k(\chi^{M-1-k})\right]}$$

we now move terms not dependent on k out of the summation:

$$= \frac{1 - e^{-\lambda}\sum_{k=0}^{\infty}\left[\lambda^k \frac{1}{k!}(1-\eta)^k\right]}{1 - e^{-\lambda}\chi^{M-1}\sum_{k=0}^{\infty}\left[\lambda^k \frac{1}{k!}(1-\eta)^k\chi^{-k}\right]}$$

and combine terms raised to exponent k:

$$= \frac{1 - e^{-\lambda}\sum_{k=0}^{\infty}\left[(\lambda(1-\eta))^k \frac{1}{k!}\right]}{1 - e^{-\lambda}\chi^{M-1}\sum_{k=0}^{\infty}\left[\left(\frac{\lambda(1-\eta)}{\chi}\right)^k \frac{1}{k!}\right]}$$

We now multiply the summation in the numerator by one, using terms such that we arrive at a new specification of the Poisson probability density function, this time with the rate parameter redefined as $\lambda(1-\eta)$:

$$= \frac{1 - e^{-\lambda}\sum_{k=0}^{\infty}\left[(\lambda(1-\eta))^k \frac{1}{k!}\left(\frac{e^{-\lambda(1-\eta)}}{e^{-\lambda(1-\eta)}}\right)\right]}{1 - e^{-\lambda}\chi^{M-1}\sum_{k=0}^{\infty}\left[\left(\frac{\lambda(1-\eta)}{\chi}\right)^k \frac{1}{k!}\right]}$$

$$= \frac{1 - \frac{e^{-\lambda}}{e^{-\lambda(1-\eta)}}\sum_{k=0}^{\infty}\left[(\lambda(1-\eta))^k \left(e^{-\lambda(1-\eta)}\right)\frac{1}{k!}\right]}{1 - e^{-\lambda}\chi^{M-1}\sum_{k=0}^{\infty}\left[\left(\frac{\lambda(1-\eta)}{\chi}\right)^k \frac{1}{k!}\right]}$$

We now repeat this process in the denominator, but with a rate parameter of $\lambda(1-\eta)/\chi$:

$$= \frac{1 - \frac{e^{-\lambda}}{e^{-\lambda(1-\eta)}}\sum_{k=0}^{\infty}\left[(\lambda(1-\eta))^k \left(e^{-\lambda(1-\eta)}\right)\frac{1}{k!}\right]}{1 - e^{-\lambda}\chi^{M-1}\sum_{k=0}^{\infty}\left[\left(\frac{\lambda(1-\eta)}{\chi}\right)^k \frac{1}{k!}\left(\frac{e^{-\lambda(1-\eta)/\chi}}{e^{-\lambda(1-\eta)/\chi}}\right)\right]}$$

$$= \frac{1 - \frac{e^{-\lambda}}{e^{-\lambda(1-\eta)}}\sum_{k=0}^{\infty}\left[(\lambda(1-\eta))^k \left(e^{-\lambda(1-\eta)}\right)\frac{1}{k!}\right]}{1 - \frac{e^{-\lambda}\chi^{M-1}}{e^{-\lambda(1-\eta)/\chi}}\sum_{k=0}^{\infty}\left[\left(\frac{\lambda(1-\eta)}{\chi}\right)^k \left(e^{-\lambda(1-\eta)/\chi}\right)\frac{1}{k!}\right]}$$

$$= \frac{1 - \frac{e^{-\lambda}}{e^{-\lambda(1-\eta)}}}{1 - \frac{e^{-\lambda}\chi^{M-1}}{e^{-\lambda(1-\eta)/\chi}}}$$

$$= \frac{1 - e^{-\lambda+\lambda-\lambda\eta}}{1 - (\chi^{M-1})e^{-\lambda+\frac{\lambda}{\chi}-\frac{\lambda\eta}{\chi}}}$$

$$= \frac{1 - e^{-\lambda\eta}}{1 - (\chi^{M-1})e^{\lambda(\frac{1-\eta}{\chi}-1)}}$$

Finally, we rewrite λ in terms of the sampling fraction (ρ) and R_{pop} as defined above, where $\lambda = \rho(R_{\text{pop}}+1)$:

$$= \frac{1 - e^{-\rho(R_{\text{pop}}+1)\eta}}{1 - (\chi^{M-1})e^{\rho(R_{\text{pop}}+1)(\frac{1-\eta}{\chi}-1)}}$$

As a check on the formulation in the equation above, let χ equal one, indicating perfect specificity of the linkage criteria:

$$\Pr(y_{ij} \mid z_{ij}) = \frac{1 - e^{-\rho(R_{\text{pop}}+1)\eta}}{1 - (\chi^{M-1})e^{\rho(R_{\text{pop}}+1)(\frac{1-\eta}{1}-1)}} = \frac{1 - e^{-\rho(R_{\text{pop}}+1)\eta}}{1 - e^{-\rho(R_{\text{pop}}+1)\eta}} = 1 \tag{12}$$

With the assumption of perfect specificity (and our original assumption that the linkage criteria identifies only a single link for a given infection), we find that any identified links will be correct. This is because perfect specificity ensures

that all negative links will be correctly avoided, leaving only true infectors as possible links.

Therefore, we can conclude that the probability of transmission given linkage, assuming single linkage and assuming k is poisson-distributed, is:

$$\Pr(y_{ij} \mid z_{ij}) = \frac{1 - e^{-\rho(R_{\text{pop}}+1)\eta}}{1 - (\chi^{M-1})e^{\rho(R_{\text{pop}}+1)(\frac{1-\eta}{\chi}-1)}} \tag{13}$$

C.3 Calculating the expected number of pairs in the sample

To calculate the expected number of pairs in the sample under the current assumptions, we start by defining the vector k_i, which gives the number of true transmission partners of infection i in a sample of size M (note that this includes the infector of i, as well us any infectees). We then define K as the summation of k_i over all i infections in the sample:

$$K = \sum_{i=1}^{M} k_i$$

Therefore, the total number of true pairs in the sample is $\frac{K}{2}$, where K is divided by two because each pair will be counted exactly twice (once as an infector, and once as an infectee, since we do not account for directionality). Accounting for the probability that a true transmission pair is correctly identified by the linkage criteria (η), the expected number of true pairs in the sample is:

$$\mathbb{E}[\text{number of true pairs}] = \mathbb{E}\left[\frac{K\eta}{2}\right] = \frac{\eta}{2} \times \mathbb{E}[K].$$

Under our assumption that each k is Poisson distributed with rate $\lambda = \rho(R_{\text{pop}} + 1)$, the sum of all k is also Poisson distributed with rate $M \times \lambda$.

$$K \sim \text{Poisson}(M\rho(R_{\text{pop}} + 1))$$

Since the expected value of a Poisson distributed discrete random variable is simply the rate λ, $M\rho(R_{\text{pop}}+1)$ substitutes for K in the expected number of true pairs.

$$\mathbb{E}[\text{number of true pairs}] = \frac{\eta}{2} \times \mathbb{E}[K] = \frac{M\rho(R_{\text{pop}} + 1)\eta}{2}$$

We can then use this to calculate the expected number of observed pairs in the sample, substituting Equation 13 for the probability an observed pair is true:

$$\mathbb{E}[\text{number of pairs observed}] = \frac{\mathbb{E}[\text{number of true pairs}]}{\Pr(\text{an observed pair is true})}$$

$$= \frac{\left[\frac{M\rho(R_{\text{pop}}+1)\eta}{2}\right]}{\left[\frac{1-e^{-\rho(R_{\text{pop}}+1)\eta}}{1-(\chi^{M-1})e^{\rho(R_{\text{pop}}+1)(\frac{1-\eta}{\chi}-1)}}\right]}$$

$$= \frac{\big(M\rho(R_{\text{pop}}+1)\eta\big)\big(1-(\chi^{M-1})e^{\rho(R_{\text{pop}}+1)(\frac{1-\eta}{\chi}-1)}\big)}{2\big(1-e^{-\rho(R_{\text{pop}}+1)\eta}\big)}$$

Therefore, the expected number of pairs observed, assuming that the number of transmission links of any infection i is Poisson-distributed and single linkage, is:

$$\mathbb{E}[\text{number of pairs observed}] = \frac{M}{2}\left[\frac{\eta\rho(R_{\text{pop}}+1)\big(1-(\chi^{M-1})e^{\rho(R_{\text{pop}}+1)(\frac{1-\eta}{\chi}-1)}\big)}{1-e^{-\rho(R_{\text{pop}}+1)\eta}}\right] \tag{14}$$

D Multiple links and multiple true transmissions

D.1 Assumptions

Here we relax the final assumption that the linkage criteria only identifies pairs of samples, and allow the linkage criteria to identify multiple links of infection i. We do, however, assume that linkage events are independent of one another, i.e. linkage of i to j has no bearing on linkage of infection i to any other sampled infection.

D.2 Derivation of the probability of transmission given linkage

Derivation for a given value of k

We begin as we did in section C.2, by deriving the probability of transmission for a given value of k, where k is the number of infections in the sample M that are true transmission partners of infection i.

$$\Pr(y_{ij} \mid z_{ij}, k) = \frac{\Pr(y_{ij}, z_{ij}, k)}{\Pr(z_{ij}, k)}$$

$$= \frac{\Pr(y_{ij}, z_{ij}, k)}{\Pr(y_{ij}, z_{ij}, k) + \Pr(\neg y_{ij}, z_{ij}, k)}$$

$$= \frac{\Pr(y_{ij}, z_{ij} \mid k) \Pr(k)}{\Pr(y_{ij}, z_{ij} \mid k) \Pr(k) + \Pr(\neg y_{ij}, z_{ij} \mid k) \Pr(k)}$$

$$= \frac{\Pr(y_{ij}, z_{ij} \mid k)}{\Pr(y_{ij}, z_{ij} \mid k) + \Pr(\neg y_{ij}, z_{ij} \mid k)}$$

Without the single linkage assumption, the probability $\Pr(y_{ij}, z_{ij} \mid k)$ is no longer simply 1 minus the probability of not linking to any true links. Therefore, we continue the derivation by applying Bayes rule and the law of total probability to each term:

$$= \frac{\Pr(z_{ij} \mid y_{ij}, k) \Pr(y_{ij} \mid k)}{\Pr(z_{ij} \mid y_{ij}, k) \Pr(y_{ij} \mid k) + \Pr(z_{ij} \mid \neg y_{ij}, k) \Pr(\neg y_{ij} \mid k)}$$

Given our assumption of independence, the probability that the linkage criteria correctly links infections i and j (i.e., $\Pr(z_{ij} \mid y_{ij}, k)$) is the sensitivity of the linkage criteria (η). And the probability that j is a transmission partner of i is simply the number of true partners of i in the sample (k), over the total number of other infections in the sample ($M - 1$):

$$= \frac{\eta \frac{k}{M-1}}{\eta \frac{k}{M-1} + \Pr(z_{ij} \mid \neg y_{ij}, k) \Pr(\neg y_{ij} \mid k)}$$

Similarly, the probability of linking i and j given that they are not a true transmission pair ($\Pr(z_{ij} \mid \neg y_{ij}, k)$) is simply the false positive rate, or $(1 - \chi)$. And the probability that j is not a transmission partner of i is the number of infections not connected to i $(M - k - 1)$ over the number of other infections in the sample $(M - 1)$:

$$= \frac{\eta \frac{k}{M-1}}{\eta \frac{k}{M-1} + (1-\chi)\frac{M-k-1}{M-1}}$$

$$= \frac{\eta k}{\eta k + (1-\chi)(M-k-1)}$$

Therefore, the probability of transmission given linkage for a given value of k is:

$$\Pr(y_{ij} \mid z_{ij}) = \frac{\eta k}{\eta k + (1-\chi)(M-k-1)} \tag{15}$$

Derivation for all possible values of k

We can extend Equation 15 to include all possibilities of k for a given infection i, again starting as in the previous section:

$$\begin{aligned}
\Pr(y_{ij} \mid z_{ij}) &= \sum_{k=0}^{\infty} \Pr(y_{ij} \mid z_{ij}, k) \Pr(k \mid z_{ij}) \\
&= \sum_{k=0}^{\infty} \Pr(y_{ij} \mid z_{ij}, k) \frac{\Pr(z_{ij} \mid k) \Pr(k)}{\Pr(z_{ij})} \\
&= \frac{1}{\Pr(z_{ij})} \sum_{k=0}^{\infty} \Pr(y_{ij} \mid z_{ij}, k) \Pr(z_{ij} \mid k) \Pr(k) \\
&= \frac{1}{\Pr(z_{ij})} \sum_{k=0}^{\infty} \Pr(y_{ij}, z_{ij} \mid k) \Pr(k) \\
&= \frac{1}{\sum_{k=0}^{\infty} \Pr(z_{ij} \mid k) \Pr(k)} \sum_{k=0}^{\infty} \Pr(y_{ij}, z_{ij} \mid k) \Pr(k) \\
&= \frac{1}{\sum_{k=0}^{\infty} [\Pr(y_{ij}, z_{ij} \mid k) + \Pr(\neg y_{ij}, z_{ij} \mid k)] \Pr(k)} \sum_{k=0}^{\infty} \Pr(y_{ij}, z_{ij} \mid k) \Pr(k) \\
&= \frac{\sum_{k=0}^{\infty} \Pr(k) \Pr(y_{ij}, z_{ij} \mid k)}{\sum_{k=0}^{\infty} \Pr(k) [\Pr(y_{ij}, z_{ij} \mid k) + \Pr(\neg y_{ij}, z_{ij} \mid k)]} \\
&= \frac{\sum_{k=0}^{\infty} \Pr(k) \eta k}{\sum_{k=0}^{\infty} \Pr(k) [\eta k + (1-\chi)(M-k-1)]}
\end{aligned}$$

Therefore, the probability of transmission given linkage for all possible values of k transmission partners in the sample is:

$$\Pr(y_{ij} \mid z_{ij}) = \frac{\sum_{k=0}^{\infty} \Pr(k)\eta k}{\sum_{k=0}^{\infty} \Pr(k)[\eta k + (1-\chi)(M-k-1)]} \tag{16}$$

Derivation if k is poisson-distributed

As in the previous section, we calculate the probability of transmission assuming k is poisson-distributed with mean $\lambda = \rho(R_{\text{pop}} + 1)$:

$$\Pr(y_{ij} \mid z_{ij}) = \frac{\sum_{k=0}^{\infty} \Pr(k)\eta k}{\sum_{k=0}^{\infty} \Pr(k)[\eta k + (1-\chi)(M-k-1)]}$$

We then pull all terms not containing k out of the sums and expand out all additions and subtractions:

$$= \frac{\eta \sum_{k=0}^{\infty} \Pr(k)k}{\eta \sum_{k=0}^{\infty} \Pr(k)k + (1-\chi)[M \sum_{k=0}^{\infty} \Pr(k) - \sum_{k=0}^{\infty} \Pr(k)k - \sum_{k=0}^{\infty} \Pr(k)]}$$

We know that the sum of a random variable times the probability of that variable is equal to the expectation of that variable, i.e. $\mathbb{E}[k] = \sum \Pr(k)k$, and that the sum of the probability of a random variable is equal to one:

$$= \frac{\eta \mathbb{E}[k]}{\eta \mathbb{E}[k] + (1-\chi)(M - \mathbb{E}[k] - 1)}$$

We also know that the expectation of a Poisson-distributed variable is equal to the rate parameter, λ:

$$= \frac{\eta\lambda}{\eta\lambda + (1-\chi)(M-\lambda-1)}$$

Finally, we rewrite λ in terms of the sampling fraction (ρ) and the effective reproductive number (R_{pop}):

$$= \frac{\eta\rho(R_{\text{pop}}+1)}{\eta\rho(R_{\text{pop}}+1) + (1-\chi)(M - \rho(R_{\text{pop}}+1) - 1)}$$

Therefore, the probability of transmission given linkage assuming k is Poisson-distributed is:

$$\Pr(y_{ij} \mid z_{ij}) = \frac{\eta\rho(R_{\text{pop}}+1)}{\eta\rho(R_{\text{pop}}+1) + (1-\chi)(M - \rho(R_{\text{pop}}+1) - 1)} \tag{17}$$

D.2.1 Calculating the expected number of pairs in the sample

To calculate the expected number of pairs in the sample allowing for multiple transmissions and multiple linkages, we start, as in section C.3 by defining K as the summation of k_i over all i infections in the sample of size M:

$$K = \sum_{i=1}^{M} k_i$$

Therefore, the total number of true pairs in the sample is $\frac{K}{2}$ and the expected number of true pairs in the sample is:

$$\mathbb{E}[\text{number of true pairs}] = \mathbb{E}\left[\frac{K\eta}{2}\right] = \frac{\eta}{2} \times \mathbb{E}[K].$$

Under our assumption that each k is Poisson distributed with rate $\lambda = \rho(R_{\text{pop}} + 1)$, the sum of all k is also Poisson distributed with rate $M \times \lambda$. Therefore, $\mathbb{E}[K] = M \times \lambda = M\rho(R_{\text{pop}} + 1)$:

$$\mathbb{E}[\text{number of true pairs}] = \frac{\eta}{2} \times \mathbb{E}[K] = \frac{M\rho(R_{\text{pop}} + 1)\eta}{2}$$

We can then use this to calculate the expected number of observed pairs in the sample, substituting Equation 17 for the probability an observed pair is true:

$$\begin{aligned}
\mathbb{E}[\text{number of pairs observed}] &= \frac{\mathbb{E}[\text{number of true pairs}]}{\Pr(\text{an observed pair is true})} \\
&= \frac{\left[\frac{M\rho(R_{\text{pop}}+1)\eta}{2}\right]}{\left[\frac{\eta\rho(R_{\text{pop}}+1)}{\eta\rho(R_{\text{pop}}R+1)+(1-\chi)(M-\rho(R_{\text{pop}}+1)-1)}\right]} \\
&= \frac{[M\rho(R_{\text{pop}}+1)\eta][\eta\rho(R_{\text{pop}}+1)+(1-\chi)(M-\rho(R_{\text{pop}}+1)-1)]}{2\eta\rho(R_{\text{pop}}+1)} \\
&= \frac{M}{2}[\eta\rho(R_{\text{pop}}+1)+(1-\chi)(M-\rho(R_{\text{pop}}+1)-1)]
\end{aligned}$$

Therefore, the expected number of pairs observed assuming that the number of transmission links of any infection i is Poisson-distributed is:

$$\mathbb{E}[\text{number of pairs observed}] = \frac{M}{2}[\eta\rho(R_{\text{pop}}+1)+(1-\chi)(M-\rho(R_{\text{pop}}+1)-1)] \tag{18}$$
