# Supplementary material for: Sample size calculation for phylogenetic case linkage
Source: PLoS Comput Biol. 2021 Jul 6;17(7):e1009182. doi: 10.1371/journal.pcbi.1009182 (PMC8284614; doi:10.1371/journal.pcbi.1009182)
Supplement: S2 Text — (PDF) [file pcbi.1009182.s016.pdf]

Text S2

Determining the sensitivity and specificity of genetic distance as a linkage criteria

A Estimating sensitivity and specificity from pathogen-specific parameters

The sensitivity and specificity of the criteria used to distinguish between linked and unlinked pathogen infections are key to determining the overall accuracy of this criteria. Here we estimate these parameters for a specific genetic distance threshold, i.e., a particular number of mutations between two pathogen sequences.

If the genetic distance distribution for linked and unlinked infections is known, determining the sensitivity (the true positive rate) and specificity (the true negative rate) for a specific threshold is straightforward and can be visualized on the distributions below:

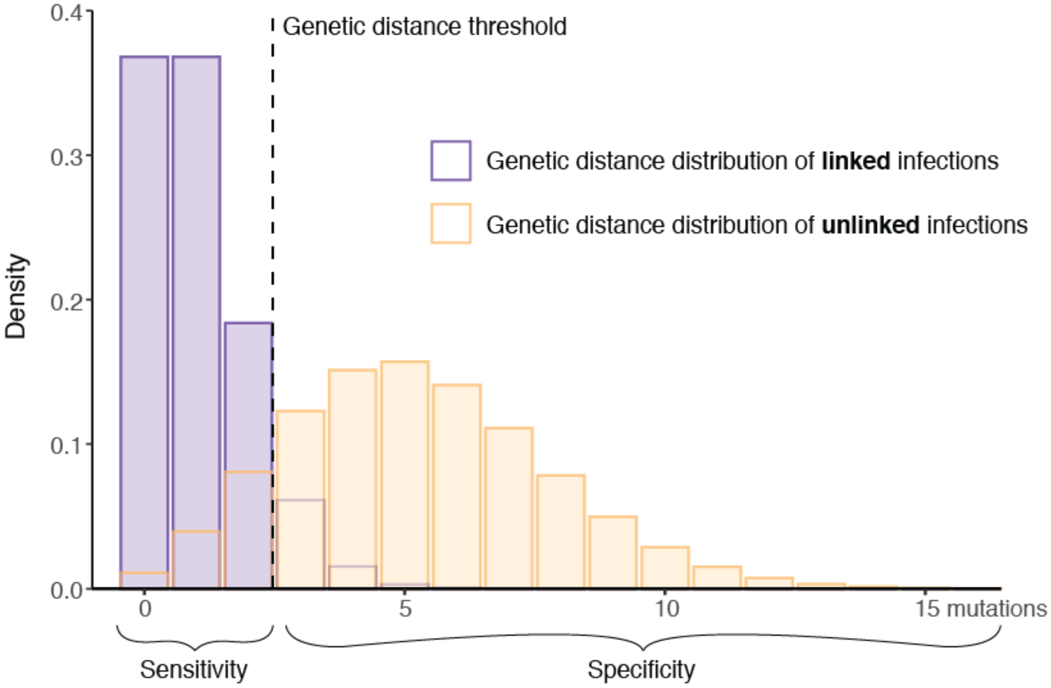

Assuming the distributions are normalized such that the total area under each curve is equal to 1, the sensitivity is simply the portion of the genetic distance distribution for linked infections to the left of the threshold (cumulative distribution function (CDF) at this threshold), and the specificity is the portion of the genetic distance distribution for unlinked infections to the right of the threshold (1-CDF at this genetic distance threshold).

The genetic distance distributions for linked and unlinked infections depend on the following:

1. The number of mutations that occur in one generation of pathogen transmission. We assume this is Poisson-distributed around the pathogen mutation rate, μ, in mutations per genome per generation.

2. The distribution of the number of generations between all infections in the population.

3. The number of generations allowed between infections considered linked. When considering direct transmissions, only 1 generation of pathogen transmission can occur between linked infections.

A.1 Deriving the genetic distance distribution for linked infections

To determine the genetic distance distribution for linked infections, we first consider the probability of observing a specific genetic distance, d between the sequences of two infected individuals linked by transmission:

$$\sum_{i=1}^{g_{\text{link}}} \Pr(\text{infections are } i \text{ generations apart}) \cdot \Pr(\text{observing } d \text{ mutations} \mid \text{infections are } i \text{ generations apart})$$
$$= \sum_{i=1}^{g_{\text{link}}} \mathrm{g}(i) \cdot \mathrm{f}(d; i \cdot \mu)$$

where g_{link} is the maximum number of generations between two linked infections.

To ensure we obtain a proper distribution (i.e., the sum of these probabilities over all values of d is equal to one), we must normalize each probability by the sum over all values of d:

$$\Pr(\text{linked infections are } d \text{ mutations apart}) =$$
$$= \frac{1}{\sum_{d=0}^{\infty} \sum_{i=1}^{g_{\text{link}}} \mathrm{g}(i) \cdot \mathrm{f}(d; i \cdot \mu)} \sum_{i=1}^{g_{\text{link}}} \mathrm{g}(i) \cdot \mathrm{f}(d; i \cdot \mu)$$

because $\mathrm{f}(d; i \cdot \mu)$ is probability density function of a Poisson distribution with mean $i \cdot \mu$, the sum of this expression over all values of d is, by definition, one. Therefore, we can simplify this equation as follows:

$$= \frac{1}{\sum_{i=1}^{g_{\text{link}}} \mathrm{g}(i)} \sum_{i=1}^{g_{\text{link}}} \mathrm{g}(i) \cdot \mathrm{f}(d; i \cdot \mu)$$

By evaluating the above expression for all values of d, we can obtain the complete genetic distance distribution for linked infections in the population, where infections are considered linked if they are separated by no more than g_{link} generations.

A.2 Deriving the genetic distance distribution for unlinked infections

We repeat the derivation above for unlinked infections, this time summing from $g_{\text{link}} + 1$ to g_{max}, the maximum number of generations considered (often equal to 2 $\times$ the duration of the outbreak, in generations of transmission):

$$\Pr(\text{unlinked infections are } d \text{ mutations apart}) =$$
$$= \frac{1}{\sum_{i=g_{\text{link}}+1}^{g_{\text{max}}} \mathrm{g}(i)} \sum_{i=g_{\text{link}}+1}^{g_{\text{max}}} \mathrm{g}(i) \cdot \mathrm{f}(d; i \cdot \mu)$$

B Simulating genetic distance distributions

As shown above, estimating sensitivity and specificity is straightforward once the genetic distance distributions of linked and unlinked infections are obtained. While the number of mutations per generation can reasonably be assumed to be Poisson-distributed, the Poisson distribution is not a good approximation for the distribution of generations between all infections in a finite population.

Determining the distribution of the mean number of generations between infections is far from trivial. Through outbreak simulations, we determined that this distribution is highly dependent on the reproductive number R of the pathogen and, to some extent, the number of generations of transmission d. Given these observations, we calculated the distribution empirically for discrete values of R between 1.3 and 18 by performing 1000 outbreak simulations for each R and averaging the distribution of generations between all pairs of infections over all simulations.

We simulated outbreaks using the *simOutbreak* function implemented in the outbreaker R package by Jombart et al. and each simulation was run for the number of generations needed to achieve a final outbreak size of approximately 1000 infections ($ln(1000)/ln(R)$), since this was the number of generations used in simulations throughout this paper. As in other simulations described in **Methods**, we we assumed a large number of susceptible individuals in the population (n.hosts=100,000) and no importation events (single source outbreak). We also assumed every infected individual transmitted their infection exactly one time step after infection (generation time = 1 time step).

The resulting averaged generation distributions are available at https://github.com/HopkinsIDD/phylosamp. Since these simulations are time consuming, especially for low values of R (which require a larger number of generations d to achieve at least 1000 infections), we used these average results when calculating the sensitivity and specificity using the mutation rate method, and provide them for others wishing to conduct similar analyses. We also note that, for a single source outbreak, the maximum possible interesting value of $g_{\max}$ is $2d$. Therefore, all distributions include probabilities for generations between infections of up to 52 generations, which is two times the number of generations (26) used in outbreak simulations for $R = 1.3$.
