# Supplementary material for: Sample size calculation for phylogenetic case linkage
Source: PLoS Comput Biol. 2021 Jul 6;17(7):e1009182. doi: 10.1371/journal.pcbi.1009182 (PMC8284614; doi:10.1371/journal.pcbi.1009182)
Supplement: Attachment — Submitted filename: phylosamp_elife_reviewerresponse.pdf [file pcbi.1009182.s017.pdf]

Response to eLife Reviewers

Reviewer #1

This paper looks at the false discovery rate in genomic epidemiology studies aiming to identify transmission pairs. This field hasn't focused much on sample size calculations or on the impact that sampling may have on inferences. It is an important topic. My major concerns are:

(1) The user needs to separately estimate sensitivity and specificity of the linkage definition that is in place. If I'm a user, once I have these, I have a lot of information. Do I need the FDR as well? The motivation is to provide a framework for study design; it might help if the authors gave an optimal study design for a hypothetical experiment and illustrated how the framework is used to obtain this study design.

We appreciate this reviewer acknowledging the importance of this work, and their very detailed investigation of some of the methods. However, we believe this concern (as well as the concerns below) is addressed later in the manuscript. In the section 'Determining sensitivity and specificity' (page 6) we provide a method for estimating the sensitivity and specificity of the linkage criteria from the data, as well as equations/code for calculating these values from the mutation rate of the pathogen. We also address this challenge at some length in the discussion. The FDR is calculated directly from these values, as shown in Figures 3 and 4 (formerly Figures 5 and 6).

We also provided a hypothetical example in one of the vignettes included in our *phylosamp* R package. However, we agree the application of our methodology could be more clear, and **we have updated our manuscript to include a worked example at the end of the 'Determining sensitivity and specificity' section.**

The following worked example was added to the 'Determining sensitivity and specificity' section: "*Regardless of which method we choose, we can use the sensitivity and specificity values to calculate the probability of correctly identifying a true transmission pair (ϕ) for this pathogen. We use Equation 1, allowing for each infection to have multiple transmission partners. We will also assume that we are able to sample 50% of the cases in this hypothetical outbreak of 1500 infections:*

$$\begin{aligned}\phi &= \frac{\eta\rho(R_{\text{pop}}+1)}{\eta\rho(R_{\text{pop}}+1)+(1-\chi)(M-\rho(R_{\text{pop}}+1)-1)} \\ &= \frac{0.98*0.5*(1+1)}{0.98*0.5*(1+1)+(1-0.99)(750-0.5*(1+1)-1)} \\ &= 0.116\end{aligned}$$

We note that, despite a reproductive number (R) of 2, a single introduction into this outbreak means we should use Rpop=1. Given our assumptions, we find that under 12% of our inferred linked infections—using a genetic distance threshold of 4 mutations—are likely to reflect true transmission relationships. A better specificity value is needed to achieve more confidence in direct transmission links, which can occur for pathogens that incur a significant number of mutations between infections considered linked [32]. For pathogens that do not meet these criteria (as in the example here), it may not be possible to use genetic distance alone to distinguish between linked and unlinked infections (Fig S4)."

> (2) I have some concerns about the methodology and derivations. The single linkage assumption is a strong one. Why would a SNP or genetic distance criterion meet this assumption? The text on page 6 centres on Rpop and the fact that if a case has an infector and some infectees then there is more than one transmission link per individual, but it does not much discuss the behaviour of real linkage definitions (though there is some derivation in the supplement).

We agree that the single linkage assumption is a strong one, and therefore this assumption is used only in the very first stage of the derivation of our method. After presenting the general framework, we relax this assumption: all later derivations do not make this assumption. In the ‘Multiple links and multiple true transmissions’ section, we address what real linkage looks like: “we will be interested in linking an infected individual to both their infector and anyone they infect. Therefore, we must account for the fact that each infection in an outbreak may be linked by transmission to multiple other infections, only some of which may have been sampled.” We believe that the reviewer’s comments are already well addressed in this section.

To avoid future confusion, **we have rearranged the manuscript to first present the ‘Multiple links and multiple true transmissions’ section**, and then to present the single linkage case as a special case afterwards. The supplementary derivations in Text S1 are still ordered from most (single linkage and single transmission) to least (multiple links and multiple true transmissions) assumptions.

> My concerns were that the chi^(M-1) and chi^(M-2) assume an independence that can't be met by the single linkage condition (see below); the statements that the number of pairs are N/2 (section A3) - surely the number of possible pairs is N choose 2 = (1/2 N (N-1)) . The number of pairs under single linkage is N/2 but the whole population of infection can't be in this single linkage mode because there have to be transmission chains in order to get N infected individuals, unless there were precisely N/2 distinct introductions. I find the derivations confusing for this kind of reason, throughout.

The reviewer is correct that the single linkage mode is not realistic for an entire population. The assumption that the number of pairs is equal to N/2 is not included in the derivations in parts C and D of Text S1.

> In section A.2 (supplement), why would P(y_{ij}, z_{ij} | S_i) be equal to 1?

P(y_{ij}, z_{ij} | S_i) is equal to one in this section because of our assumption of perfect sensitivity. As stated in the assumptions section, “The sensitivity of the linkage criteria is equal to 1 when both the infector and infectee have been sampled. If infection i is truly linked by transmission to infection j and both infections are found in sample M , then y_{ij} = 1 by definition. Under this assumption of perfect sensitivity, z_{ij} = 1 as well.”

Given perfect sensitivity, the linkage criteria will always correctly pair infection i to its true pair if both have been sampled. We note that this assumption is relaxed after section A of Text S1, and we no longer assume this expression is equal to 1.

> I derived equation (1) for phi by noting that the denominator is composed of all the ways a link can turn up, and the numerator is the term for these that are true. This relies on, for example, 1- chi^{M-1} meaning not (all unlinked) --> at least one is linked, which in turn uses an independence assumption that isn't met by a linkage

system that guarantees precisely one link for each case. I suspect that this carries through the more detailed derivation in the appendix.

This independence assumption is implied by our assumption that every infection has been connected by transmission to exactly one other infection. However, we once again note that this assumption is only required for the single linkage, single transmission special case, and does not carry through to the more general and more broadly applicable multiple transmission scenario. Therefore, this concern does not affect our main derivation or the validation of our method in any way.

The FDR is defined as the portion linked that are not transmission pairs, or "linked and not transmission / total linked". The probability of correctly identifying a true transmission pair intuitively seems to be (linked and true pairs) / (true pairs). The denominator differs between these two. This is perhaps just due to imprecise language but it seems important.

In the manuscript, we define the false discovery rate as 1 – "the probability than an identified link represents a true transmission event". In other words we define the false discovery rate as 1 – P(y_{ij} | z_{ij}). In our derivation, this is described as 1 – (linked true pairs / (linked true pairs + linked false pairs)). This is equivalent to saying that the FDR is equivalent to "linked and not transmission / total linked", as the reviewer correctly states. We do in fact want "total linked" in the denominator because we are concerned with the FDR of the linkage criteria, and have confirmed that this is the only way we define the FDR in the manuscript.

Line 290: "conditional mean" - conditional on what?

This comment refers to the Figure 3 (formerly Figure 5) legend. Here, smoothed conditional mean refers to the mean simulated false discovery rate conditional on the x-axis (the theoretical FDR). We are using the geom_smooth function from the R package ggplot2 to calculate this, and have updated our methods to reflect this detail. **The 'Outbreak simulations' section of our methods now contains the additional information:** *"Validation plots were made in R using ggplot2 [33], and smoothed conditional means were calculated with the geom_smooth function from this package."*

Figures 5, 6, S6: what is N?

In each figure legend, we state that these are the results of 10,000 simulations. However, we understand that the reviewer may be asking about the number of points in each plot. **We have updated each legend with the total number of points, and have also included the range of N (meaning final outbreak size) values for each simulation.** (Note that Figures 5, 6, and S6 are now Figures 3, 4, and S8.)

The figure legends now contain: *"Theoretical versus simulated false discovery rate (FDR) for each genetic distance threshold in 10,000 simulations of varying mutation rate and reproductive number (approximately 260,000 points per plot, see Table 2). Outbreak sizes range from 100–2000, as described in Methods."*

What is the story about the horizontal lines of estimates at a simulated FDR of 0 and 1? But theoretical FDR ranging from 0 to 1?

Good question! This is an artifact of simulating outbreaks: the horizontal line at simulated FDR = 0 occurs when, by chance, there are no false positives in our simulation. In other words, there are no incorrectly-linked transmission pairs. This can occur when our genetic distance threshold is very low (e.g., 0 mutations). In this case, very few pairs of infections will be identified as linked, and it is possible that all pairs of samples with identical (simulated) genomes are in fact transmission links. Since the FDR is "linked and no transmission / total linked", no occurrences of the "linked and no transmission" scenario will result in FDR = 0.

Similarly, the horizontal line at FDR = 1 occurs when, by chance, our simulations do not capture any linked true transmission pairs. This can happen especially when the mutation rate is high, resulting in high genetic distance between true pairs. In this case, a low genetic distance threshold may produce no links between true transmission pairs. In this case, the "total linked" will be equal to "linked and no transmission", resulting in FDR = 1. Because this is due to random chance during the simulations, these lines occur only in simulated data.

We have added a short explanation of this phenomenon to the text at the bottom of page 10: "Additionally, while our method is an unbiased estimator and overall correct in expectation, it is always possible for performance in a particular set of individuals sampled from a population to deviate substantially from expectation. *As an example, in a small fraction of simulations, there were by chance no true transmission links (or, in some cases, no false positives) in our subsample. This fixes the simulated false discovery rate at 1 (or 0, when there are no false positives), which may not be representative of the overall relationship between sample size and false discovery rate and highlights how the specific infections sampled can affect results, particularly when sample sizes are low."*

> Under single linkage you need lone infector-infectee pairs and no transmission chains. How does this constraint affect the relationship between the sample size and the FDR?

Because we include the single linkage case only for purposes of illustration (recognizing that this is not a realistic scenario), we do not explore this relationship. Figures 3 and 4 (formerly Figures 5 and 6) are only created using our derivation for the multiple link, multiple transmission scenario.

> Figure S6 and similar figures - if you had just one dataset, what is the probability that you land well away from the line?

Good question! And we agree it is one users of this method may be interested in. To answer this question, **we have added two additional figures**. The new Figure 5 (page 13) is a series of histograms showing the error of each calculated FDR, sensitivity, and specificity value compared to the corresponding simulated outbreak. The new Figure S6 shows the same, but only for the optimal sensitivity and specificity thresholds (based on genetic distance as a linkage criteria). For reference, these figures are reproduced below as well. (Note that the old Figure S6 is now Figure S8, and the new figure falls into place as Figure S6.)

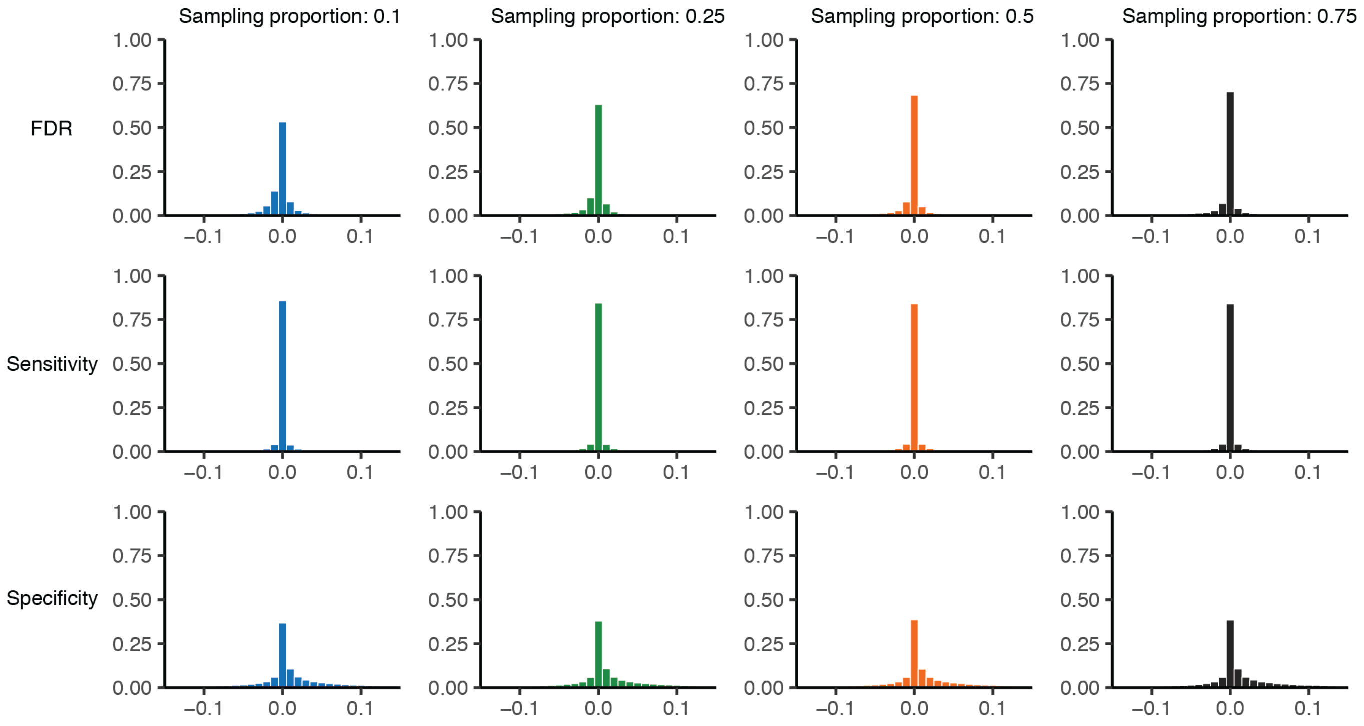

Figure 5: Histogram of raw parameter error using mutation rate method.

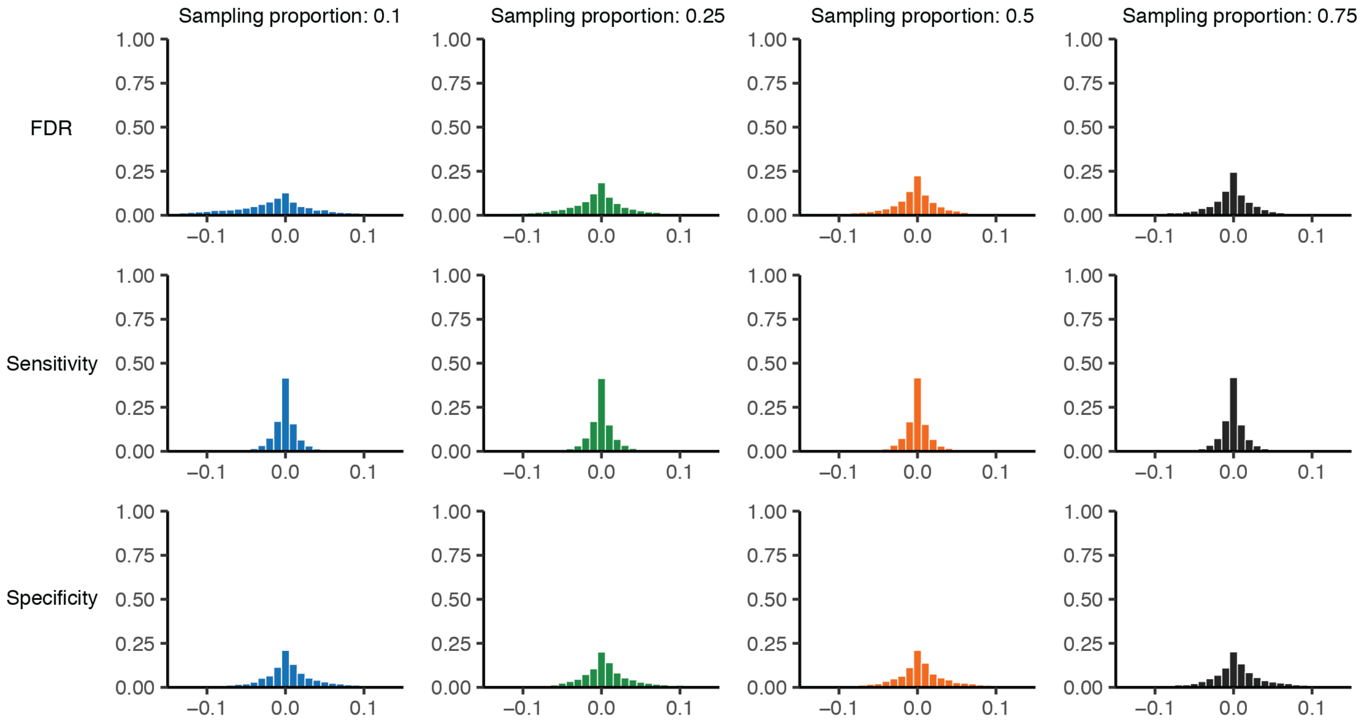

Fig S6: Histogram of raw parameter error using mutation rate method (optimal threshold only).

Reviewer #2

In this manuscript, Wohl and colleagues developed a statistical framework to help calculate the sample sizes in phylogenetic studies. Their method calculates the true discovery rate and expected number of true infector-infectee transmission pairs, based on the sensitivity and specificity of the linkage criteria, proportion of sampled infections, and sample size. They considered two possible transmission scenarios, (1) each infected person is only connected to one other person by transmission, and (2) each infected person may be linked to multiple other infections. To calculate the sensitivity and specificity of linkage criteria, the authors provided two possible methods, i.e., empirical method and mutation rate method. The topic is relevant to phylogenetic studies and the scope of eLife. The method developed is acceptable.

I have some comments and suggestions:

(1) In Table 1, the meaning of parameter R_{pop} (average reproduction number of a pathogen in a finite population) is not very clear. In page 6, the authors defined it as the average number of infectees per infector in the finite sampling population. Why it is always smaller than 1. It'll be helpful if the authors could give an illustration figure.

This is an important concept, and we have already provided an explanation in the section 'Estimating the average reproductive number' (page 6) and indeed provided Figure S1 (now, Figure S2) to clarify this complicated matter. **We have added some additional detail to the legend of this figure to clarify how R_{pop} is calculated.**

The legend now reads: "Two hypothetical outbreaks with a pathogen reproductive number (R) equal to 2 and a total of 15 infections. Black circles represent infections; blue circles represent infections who have not yet infected others, or whose descendents are outside the sampling frame. (A) Outbreak caused by a single introduction, *meaning there were 14 transmission events and 15 total infections. In other words, resulting in Rpop=1415=0.933.* (B) Outbreak caused by two separate introductions, *meaning there were only 13 infection events in the sampling frame, resulting in Rpop=1315=0.867."*

(2) Regarding the addressed two possible transmission scenarios in pages 4 to 6, the probabilistic structure considered in the equations suggests that the transmission seems to be random. For example, an infectious contact may occur between two randomly selected people. It'll be valuable if the authors could extend their framework to address the contact network underlying a population (e.g., clustering, or hub nodes).

This is an excellent point and will be the subject of our future work in this space. This manuscript seeks to provide a basic framework starting with mass-action transmission dynamics , and we hope to extend this framework to account for these types of heterogeneities. **We have updated the last paragraph of our discussion to address this important future direction.**

This paragraph now reads: "We hope that this work represents a step towards developing a larger theory of study design for making inferences from pathogen sequence data, but recognize it is only a step. *The focus of this paper is sample size and the impact of undersampling, but spatial and/or temporal biases are also important for determining which infections are sampled [36–38].* For example, understanding routes of direct transmission may require dense sampling of a small group of highly-connected individuals, while understanding general transmission trends over the course of a geographically-dispersed outbreak may require us to sample broadly over space and time. *Additionally, it will be important to take into account the contact network underlying pathogen transmission,*

since some individuals may be more likely to transmit their infection to others. Finally, the goal of linking infections is seldom the linkages themselves, but the larger inferences about risk and transmission derived from those linkages. Adapting the techniques here to more directly link sample size calculations to these outcomes is an important next step."

(3) In page 7, the assumed mutation rate (1 mutation per genome per generation) may be too high? Probably good to give some examples of real-world diseases with this mutation rate.

This is also a fair point. Although slightly higher than an RNA virus mutation rate, we selected this value for illustrative purposes (specifically, genetic distance distributions for linked and unlinked samples are more distinctive for high mutation rate pathogens). We also note that our simulations are performed on a wide range of possible realistic mutation rates, as described in the Methods section ('Outbreak simulations'). That said, we would be happy to include additional versions of Figures 1 and 2 using additional mutation rates in the supplementary material, if the editors feel this is important.

(4) In page 8, "empirical method", which evolutionary model is used in the simulation? GTR?

We used the simOutbreak function from the *outbreaker* R package for our simulations. The evolutionary model is indeed a time-reversible Markov process where all sites mutate independently, and we selected parameters so that the transition rate was equal to the transversion rate. Since we are not working with real pathogens, we were more interested in the overall mutation rate for our calculations. **We have updated the methods section to specify this.**

The 'Outbreak simulations' section now contains: "We chose these ranges to encompass mutation rates and reproductive numbers observed in actual human pathogens, *and set the transition rate to be equal to the transversion rate for the purposes for this simulation.*"

(5) In page 9, Eq. (3), the conditional probability distribution f(d; i, \mu) may be also hard to calculate. The authors may wish to give more details or guidance on calculating this conditional distribution.

We disagree and believe this is one of the few terms that is easy to calculate. We provide details in Text S2, **but have added a note in the 'Determining sensitivity and specificity section** that f(d; i \mu) is simply the probability density function of a Poisson distribution with mean i * \mu: *"Since we assume that the number of mutations between two linked infections is Poisson distributed, f(d;i)is simply the probability density function of a Poisson distribution with mean i."*

(6) Page 10, "outbreak simulations", why you need to assume "every infected individual transmitted their infection exactly one time step after infection". The unit time step is not clearly defined.

In the *outbreaker* R package used for simulations, the time step unit is in fact not clearly defined. Instead, the user can define this unit by providing information on when each infection is transmitted. By providing a value such that "every infected individual transmitted their infection exactly one time step after infection", we are essentially stating that each time step is equivalent to the transmission generation time, and that this generation time distribution is very narrow. In other words, we are simulating simplified outbreaks in terms of temporal generation, and are concerned only with generations, not calendar time.

(7) The authors may wish to apply their theoretical framework to some real-world data. For example, data used in this paper: https://www.medrxiv.org/content/10.1101/2020.08.13.20174136v2

This is an excellent point, and something we hope to do in the future. As of now, our framework is aimed at answering questions about transmission between individuals. Applying this framework to more realistic transmission questions is not addressed in that paper, and is not something we believe the data in that paper is adequate to explore. We do hope to apply this work to more real world situations in the very near future.

Reviewer #3

Authors have developed a statistical framework for calculating the number of true infectee-infector pairs identified in the dataset, given a specific sample size and sampling fraction. They propose that their approach can conversely be used to calculate the sample size (in terms of sequences) necessary to identify pairs linked through transmission, given a pre-specified false discovery rate and sampling proportion. This method is illustrated using simulations. Their overall aim is to help guide the design, and/or evaluation of quality of results of previously conducted studies of pathogen transmission. They report that the error in estimating the false discovery rate was <4%, and decreased as sampling proportion and sample size increases. The tool developed is available open-access as an R package.

Overall assessment:

This is a well-written and clear manuscript. The method proposed is much needed in phylogenetic studies - where many have been conducted using available samples taken from storage, without consideration of the sampling and whether this was appropriate to answer the study question. One key concern I have is that, while I appreciate this is meant to introduce the methodology, it would have been helpful to see this implemented on an actual real-life dataset, rather than purely simulation alone. As it stands, this is perhaps better suited to a biostatistics or computational biology journal.

After reading the comments of this reviewer and reviewer #2, we agree that without application to a real-life dataset this manuscript may be better suited to a computational biology journal. As such, we are submitting the manuscript to PLoS Computational Biology.

Major considerations:

Authors state that 'undersampled or biased sampling can lead to poorly supported inferences about patterns of disease spread'. I absolutely agree. However, it seems that authors have really only focused on one aspect of this - undersampling - and not examined the influence of biased sampling strategies. Specifically, authors appear to assume each element has equal probability of being selected for inclusion. I wonder about the validity of this assumption; it seems more likely that samples available or selected for inclusion in a study may be biased towards those in a particular geographical region, time frame, or selected based on epi characteristics, e.g. known linkages. How will samples being available in a non-random manner affect the results of this approach?

It is true that we assume random sampling and focus on the impacts of undersampling. We also recognise that other forms of sampling bias, in particular spatial and temporal bias, are important factors that have been underexplored

to date. This is something we hope to address more fully in future iterations of this work. We bring attention to the importance of this type of sampling bias in the discussion, and **we have added some more commentary on the importance of understanding the transmission network when selecting samples for sequencing.**

Specifically, the last paragraph of the discussion now reads: “We hope that this work represents a step towards developing a larger theory of study design for making inferences from pathogen sequence data, but recognize it is only a step. *The focus of this paper is sample size and the impact of undersampling, but spatial and/or temporal biases are also important for determining which infections are sampled* [36–38]. For example, understanding routes of direct transmission may require dense sampling of a small group of highly-connected individuals, while understanding general transmission trends over the course of a geographically-dispersed outbreak may require us to sample broadly over space and time. *Additionally, it will be important to take into account the contact network underlying pathogen transmission, since some individuals may be more likely to transmit their infection to others.* Finally, the goal of linking infections is seldom the linkages themselves, but the larger inferences about risk and transmission derived from those linkages. Adapting the techniques here to more directly link sample size calculations to these outcomes is an important next step.”

> In ongoing outbreaks, particularly with slowly-mutating pathogens, there is very low genetic diversity. Using genomic data alone as the linkage criteria would prohibit detection of true infectee-infector pairs. While authors mention that epidemiological data can be used in this linkage, they do not demonstrate this in the current analysis, and I wonder about the utility of this method, given that these scenarios are when identifying the precise infectee-infector pairs, and clusters, are most needed from a public health standpoint.

This is a good point. Genomic data alone will likely not provide the needed resolution for many slow-mutating pathogens. We believe that expanding this method to linkage criteria beyond genetic distance is a crucial next step and future direction, and finding better ways to integrate epidemiological (and other) types of data will be an important part of this but is outside the scope of this introductory manuscript. We also acknowledge that identifying precise infector-infectee pairs might not be possible in all situations, and understanding when increased sampling can or cannot lead to the desired answers is an important part of developing a sampling framework.

> I wonder about the assumption of a single source in all outbreak simulations. This is unlikely to be realistic for settings with high levels of transmission or endemic disease. How would inclusion of multiple sources affect these results? Also, what is the rationale for using only 1000 nt genome length?

This is an interesting question, but would likely be better suited to another manuscript that explores additional transmission scenarios, especially since performing this type of simulation would require additional assumptions (e.g., the amount of diversity globally and how much of this diversity is imported in each simulation). While, if the editors thought it was important, we could add an illustrative simulation with multiple introductions, exploring this space more broadly would be a large endeavor that would add significantly to the complexity and length of the paper while minimally adding to the overall goal of laying out a broader framework.

The rationale for using 1000 nucleotide genome length was purely for computational speed. All mutation rates have been adjusted for this genome size, so this should not have a significant effect on our conclusions.

The idea that a sampling frame is finite - it seems like authors are assuming a closed population, but in reality, this is rare. How do authors account for edge effect, for example, wherein the source or secondary cases are outside the time period selected for study?

We have updated the wording in this section to clarify that while the population itself is open, sampling over a particular period of time/space implicitly creates a finite population: *"However, sampling infections over a finite period of time, for example, produces a bounded sampling frame."* The edge effect question is an interesting one, but we note that samples are always taken over a particular time and space, so edge effects will always exist unless you have a fully observed epidemic in a closed population. Therefore, we believe it is important to define a sampling frame such that, with enough sampling, it would be possible to answer the questions at hand. Our method only deals with what happens after the sampling frame has been established, not the types of questions that can be answered with this frame. All that said, we note that edge effects shouldn't affect the primary outcome of our work (the false discovery rate), since we are concerned with what proportion of identified links actually represent true transmissions, regardless of the sampling strategy used.

Table 2 - FDR=0.00-0.25 and sampling proportion of 0.10 - there is negative bias? Also, why does bias seem to increase for sampling proportion of 0.75 compared to 0.50 for most of the FDR ranges, when it was otherwise trending downwards as proportion increased?

The reviewer correctly points out that the bias at FDR=0.00-0.25 and sampling proportion of 0.10 is a negative number. However, at -0.0006 this value is effectively zero, so we do not consider this to be evidence of significant negative bias, especially given the comparatively low sample size of points with a FDR value in this range. Similarly, while the reviewer is correct about the reversed trend in sampling proportion, this is likely an artifact of average over many simulations, which are stochastic with many degrees of freedom. Regardless of the specific trends in bias, this table and corresponding figures strongly validate our overall approach to calculating the false discovery rate from the sensitivity, specificity, sample size, and sampling proportion.

Authors state that, using their mutation rate method to estimate sensitivity and specificity, their estimates for specificity have positive bias and large error - especially for low sample size. They refer to Fig. S6, but it would be helpful to see a figure with this at different sample sizes indicated. What, by their definition, is "low sample size"?

We appreciate the reviewer bringing this up, as this is actually a mistake. When we refer to low sample size, we refer to the lighter colors in Fig S6 (now Fig S8), or 0-25% of the maximum sample size for a given sampling proportion. The simulation size was capped at 2000 infections, so a sampling proportion of 0.50, for example, means a maximum of 1000 infections could have been sampled, and the lowest sample size category would be 0-250 infections. These are the sample size categories used in Tables S1 and S3 as well. However, further investigation into our specificity estimates (shown in Fig S6, now Fig S8) by sample size show that sample size and proportion do not have an outsize effect on the bias or raw error of the specificity. This is reflected in the histograms below, **added as Figure S9**, where the x-axis is the calculated specificity for a particular genetic distance threshold minus the true specificity for that simulation and threshold. **We have updated the text to mention only the overall bias and point to this new figure for a detailed look into how sample size and proportion affect specificity estimates using our method.**

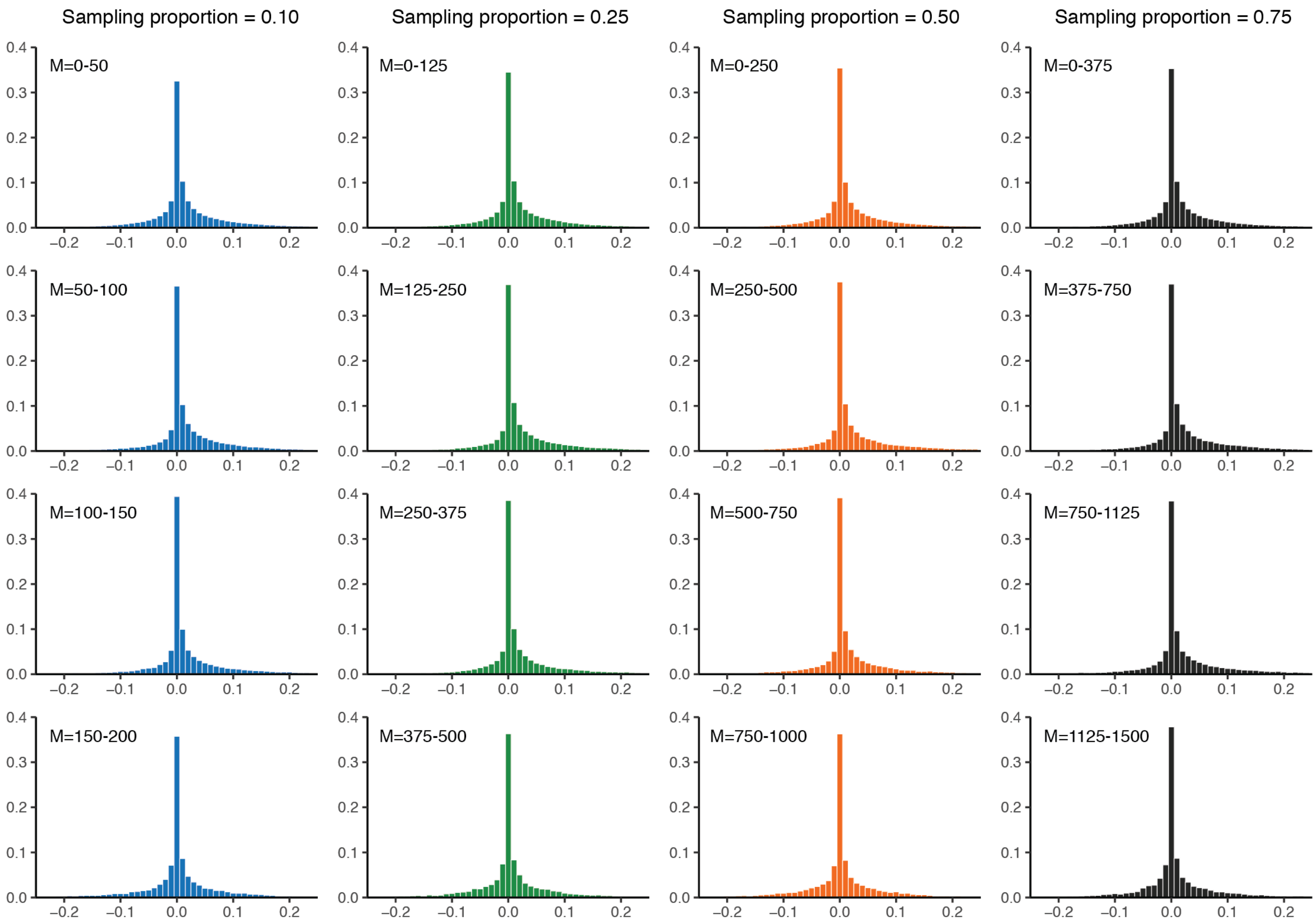

Figure S9: Histogram of raw specificity error using mutation rate method by sample size and proportion.
