# Supplementary material for: Sample size calculation for phylogenetic case linkage
Source: PLoS Comput Biol. 2021 Jul 6;17(7):e1009182. doi: 10.1371/journal.pcbi.1009182 (PMC8284614; doi:10.1371/journal.pcbi.1009182)
Supplement: Attachment — Submitted filename: phylosamp_ploscompbio_reviewerresponse.pdf [file pcbi.1009182.s018.pdf]

## Response to PLOS Computational Biology Reviewers

# Reviewer #1

Wohl et al present a statistical framework for calculating sample sizes for robust determinations of the infector-infectee pairs within transmission chains of pathogen genomic epidemiology studies. Their framework also provides methods for calculating FDR and the expected number of true transmission pairs from the specificity and sensitivity of the linkage criteria (genetic distances), sample size, the proportion of samples sequenced, and the effective reproductive number of the pathogen analysed. The authors demonstrate the utility of this framework with simulation data and developed the R package "phylosamp" to provide an implementation of their framework.

This manuscript addresses a neglected problem in many genetic epidemiology studies regarding the level of sequencing required to be carried out in order for robust conclusions to be made when reconstructing transmission chains of pathogen outbreaks using WGS data. The work is novel as there are a lack of current formal agreed upon standards for carrying out this aspect study design, and is both relevant and timely given the increasing widespread adoption of genetic epidemiology techniques for understanding pathogen transmission dynamics. Further, the manuscript is well written, the underlying methodology well described, and the use cases of the software and limitations are appropriately discussed.

Please find my comments below, divided into different sections for (a) the manuscript describing the framework and (b) the R package phylosamp. I hope these are useful to the authors.

We thank the reviewer for these helpful comments.

(1) The reliance of phylosamp at present on genetic distances alone as the linkage criteria presents a key limitation in calculating appropriate sample sizes and other parameters for a study concerning slowly evolving pathogens where there is limited genetic variation accumulating between transmission pairs/generations which prohibits their detection from WGS alone. I recognise that the focus of this manuscript is a first step towards more comprehensive approaches, and that these concerns are discussed in both the manuscript, and in previous supplied reviews from a submission to eLife, but also believe that this limits the utility of the software for many genetic epidemiology studies.

Although we agree with the sentiment that using genetic distance as a linkage criteria is just a first step, we would like to clarify that the *phylosamp* software is not limited to genetic distance. We provide genetic distance as a key example because it is a straightforward linkage criteria that lends itself to easy calculation of sensitivity and specificity. However, the equations underlying the functionality in the *phylosamp* package rely only on knowing the sensitivity and specificity of the linkage criteria, and make no requirement that the linkage criteria be genetic distance. Therefore, the software should be easily portable to other definitions of linkage. While we do not provide details on how to calculate sensitivity and specificity from other criteria, we hope to continue work in this space and hope that the guidance we provide around genetic distance is useful to researchers hoping to apply our method to other genetic epidemiology studies.

We have further emphasized this in the revised discussion section, where we state:

> **… more nuanced criteria such as phylogenetic relatedness will likely be more informative than the number of mutations between sequenced infections; while we provide instructions for using genetic**

**distance as a linkage criteria in order to give a concrete example of calculating sensitivity and specificity, the primary focus of this manuscript is to demonstrate how they can be used to calculate or evaluate sample sizes.**

(2) While the simulation data provide a useful and convincing illustration of the framework, it would be excellent to also see an example application of phylosamp to an existing published pathogen dataset to further demonstrate its utility. Again, I recognise that this has been discussed in previous reviews from a submission to eLife, but the inclusion of such data would present a substantial improvement to the work and encourage further adoption of the framework.

We appreciate this point and have added a section to the end of the results where we apply the *phylosamp* package — and the substitution rate method for calculating sensitivity and specificity, also implemented in the R package — to a published dataset. We use this to show how our method can easily demonstrate if genetic distance is an appropriate linkage criteria for distinguishing between linked and unlinked pairs:

***Illustrative retrospective example***

**To illustrate our sample size calculation framework, we used a publicly available dataset from an outbreak caused by a well characterized pathogen (mumps virus) that had been subject to both genomic and epidemiological analysis [37]. We first used the substitution rate method described above to calculate the sensitivity and specificity of genetic distance as a linkage criteria using the substitution rate reported in the study (molecular clock rate = 4.7610-4substitutions per site per year). We converted this substitution rate to 0.36 substitutions per genome per generation using the mean generation interval estimated in the study (18 days), which falls within previous estimates of this parameter [42–44]. We used the effective reproductive number reported for Harvard University (1.70) to estimate the generation time distribution using our phylosamp package, as shown in the R code below:**

```
library(phylosamp)
data("gen_dist_sim")
mgd <- as.numeric(gen_dist_sim[gen_dist_sim$R == 1.70, -(1:2)])
get_optim_roc(sens_spec_roc(cutoff=1:20,mut_rate=0.36,mean_gens_pdf=mgd))
```

**This method results in an optimal sensitivity of 0.95 and specificity of 0.95 using a cutoff of two mutations.**

**We then used these parameter values to calculate the true discovery rate of our linkage criteria, i.e., the proportion of identified links (whole mumps genomes differing by <2 mutations) that represent actual transmission pairs. We focused on the part of the mumps outbreak within Harvard university, for which 66 whole genomes sequences were generated from 71 unique patient samples. While the true number of cases at Harvard was likely significantly higher, this provides a maximum sampling proportion of 93% of infections. Using the phylosamp package, we calculated the true discovery rate as follows:**

```
truediscoveryrate(eta=optim$sensitivity,chi=1-optim$specificity,rho=0.93,M=66,R=1)
```

**Using our method, we calculated a true discovery rate of 0.35. This low value suggests that genetic distance alone would not be sufficient to identify specific transmission links within the Harvard community during this mumps outbreak. This is in line with the findings of the original paper, which demonstrates the need for both genomic and epidemiological data to understand transmission, and**

**emphasizes the frequent need for such epidemiological data to achieve the required specificity for high confidence estimation of transmission links.**

***Illustrative prospective example***

**To demonstrate how our method could be used to estimate the sample size needed to identify transmission links with 90% confidence (i.e, a true discovery rate of 0.9), we applied our method to a hypothetical COVID-19 outbreak in an unvaccinated community with 120 infections. We calculated the sensitivity and specificity of genetic distance using a substitution rate of 0.34 mutations per genome per generation [38–41] and an R value of 3, consistent with many efforts [45,46]:**

```
mgd <- as.numeric(gen_dist_sim[gen_dist_sim$R == 3, -(1:2)])
get_optim_roc(sens_spec_roc(cutoff=1:20,mut_rate=0.34,mean_gens_pdf=mgd))
```

**This method results in an optimal sensitivity of 0.95 and a specificity of 0.84 using a cutoff of two mutations. Using these parameters, we found that not even perfect sampling could lead to a true discovery rate of at least 0.9:**

```
samplesize(eta=optim$sensitivity,chi=1-optim$specificity,N=120,R=1,phi=0.9)
```

**This suggests that genetic distance alone is not sufficient to differentiate linked and unliked SARS-CoV-2 infections at high confidence. However, if we could identify additional phylogenetic or epidemiological criteria that would increase the specificity to 0.999 (keeping the sensitivity at 0.95), a sample size of 11 would achieve our desired confidence in direct transmission links. Additionally, it may be more fruitful to focus on cases linked within several generations of transmission, during which additional mutations would have time to accumulate.**

We have also added a section to the discussion that comments on the importance of a well-characterized mutation rate and pathogen reproduction number for applying our method:

**The application of our methodology to a previous mumps outbreak and a hypothetical COVID-19 outbreak highlights the need to move beyond genetic distance as a linkage criteria; for pathogens with a substitution rate similar to that of mumps virus, genetic distance is not enough to differentiate between linked and unlinked cases even in densely sampled outbreaks. In trying to apply this method to other outbreaks, it also became clear that well-characterized mutation rates and reproductive numbers are essential for calculating sensitivity and specificity using our method, and that these parameters are less clearly defined for pathogens with long and variable generation times, such as bacterial infections. Variable periods of replication within a host makes it difficult to characterize a per-generation substitution rate that is broadly applicable over the entire outbreak and can be used to estimate sensitivity and specificity. In these cases, more nuanced criteria such as phylogenetic relatedness will likely be more informative than the number of mutations between sequenced infections; while we provide instructions for using genetic distance as a linkage criteria in order to give a concrete example of calculating sensitivity and specificity, the primary focus of this manuscript is to demonstrate how they can be used to calculate or evaluate sample sizes.**

Finally, we have updated the methods to describe the details of this application to a mumps dataset.

(3) The definition of Rpop provided from line 100, where it is first introduced requires rephrasing for clarity. While this is better described later in the manuscript from line 149, the earlier text could be clarified to avoid the reader having to scroll back and forth throughout the paper. I recognise that this text has already been refined

> based on the reviewer comments from the previous submission to eLife, however, it could benefit from further refinement for improved clarity and flow.

We have continued to refine Rpop based on comments from multiple reviewers. This section now reads:

> Because we allow each infection to have multiple transmission partners, this probability will also depend on the average number of transmission links per infection, which is determined by the epidemiological parameter R, the expected number of other individuals each infected individual infects in a fully susceptible population. However, sampling infections over a finite period of time produces a bounded sampling frame, in which the average number of infectees per infector, denoted Rpop, may differ from R. **This is because terminal nodes in the transmission network within this finite sampling frame are presumed to have no known child infections, and therefore an R value of zero. These nodes (which may or may not have child infections outside the sampling frame) contribute an R value of 0, decreasing the average number of infectees per infector.** In fact, Rpop must be less than 1, see 'Estimating the average reproductive number' below.

> (4) Figure 1B: Does each white dot indicate the sensitivity and 1-specificity for a SNP/genetic distance increased in increments of 1? i.e. 0, 1, 2, 3, 4, … SNPs? If so, it would be helpful to indicate the values of these increments either by annotation of the figure itself or expansion of the figure legend to improve clarity.

We appreciate and agree with this clarifying suggestion. We have updated the figure legend to include additional information about the white dots:

> Effect of varying the sensitivity and specificity of the linkage criteria on the false discovery rate (FDR). White dots: theoretical sensitivity and specificity values at different genetic distance thresholds **(1-10 mutations between infections; leftmost white dot represents a threshold of 1 mutation)** for a hypothetical pathogen with substitution rate = 1 mutation/genome/transmission and R=2 (see 'Determining sensitivity and specificity' below for details).

> (5) Line 242: There don't appear to be any citations for the range of effective reproductive numbers of human pathogens explored in simulation studies.

We thank the reviewer for pointing this out, and have added citations for both parameters. We want to emphasize that the goal was to provide a range of realistic values and to test our methods on many combinations of R and $\mu$, not necessarily to start and end at the minimum and maximum observed values. Additionally, while it is certainly possible to have effective reproductive numbers below 1.3, we were primarily interested in exploring pathogens that caused outbreaks of a substantial size (minimum outbreak size = 100 infected individuals) so that we could perform statistics on the results. Values below 1.3 were computationally intractable because of the time it took to generate large enough outbreaks. We have clarified this in the text by adding the following sentence to the methods:

> **We note that, while pathogens can have reproductive numbers below 1.3, this was the minimum value that produced enough outbreaks with greater than 100 individuals in a reasonable amount of time.**

> (6) Figure S5: It appears that either the figure panels or the legend descriptions might be inverted for A and B, as well as C and D.

We thank the reviewer for pointing out our mistake. We have swapped A/B and C/D in the figure itself.

(7) The authors have put substantial effort into making their work openly available by submitting a preprint on medrxiv and providing all code and data files required to reproduce their analyses and manuscript figures via github (available at: https://github.com/HopkinsIDD/phylosamplesize). I was able to reproduce all figures and analyses until line #113 of figures.Rmd at which point I was unable to proceed further.

```
# first time only: calculate tfdr from simulations and save to file
calc.tfdr(simdata="data/simdata_var_N10000",rho_values=c(0.1,0.25,0.5,0.75),max_sim_size=2000,
sens_spec_method="sim",mgd=mgd,outdir="data/full_data_sim.Rdata")
```

I think this might be due to the files being specified by the prefix "simdata_var_N10000" where it might need to be instead specified as "simdata_var_gen_N10000", but the authors may need to look into this further.

Thank you for pointing out this typo. The reviewer is correct that "data/simdata_var_N10000" should be "data/simdata_var_gen_N10000". We have updated the code accordingly.

Code from the R package was clearly structured and generally well commented. The package is freely available and easily installed via the devtools library. I was able to reproduce the results from the vignette code easily and without issue, and found the explanations very clear and informative. I have provided some comments on the R package and documentation below that I hope are useful to the authors, but do not regard any of these to be critical changes, nor do I require that these suggested changes be made for the publication of this manuscript.

We thank this reviewer for their positive comments on the package and suggestions. We have made the changes suggested and they will be available in the next version of the package, which we will make available prior to publication of our manuscript.

## Reviewer #2

Wohl et al. present a method for understanding how sampling, both in terms of overall depth and in terms of proportion, influences how accurately we can identify true infector-infectee pairs (linked cases) from a phylogeny of pathogen genomes. This theoretical area of genomic epidemiology is sorely underdeveloped, especially when compared to the rigorous theoretical framework for sampling design available for traditional epidemiological studies. This work is the first real step I've seen to develop sample size calculations for genomic epidemiological studies. The manuscript is clearly written, and I am satisfied by how the authors have addressed previous reviewer comments. While this work should be accepted, I do have some minor comments that should be addressed to avoid reader confusion and position this paper in the appropriate context. These comments do not require further analytic work; they are only textual changes.

We thank this reviewer for their positive comments.

1. In the Introduction the authors draw on many examples of how pathogen genomic information can be used to investigate public health questions (lines 34-37) at multiple scales (lines 47-49), and declare that all of those questions can be boiled down to a question of asking whether pairs of infections are related. I disagree with this,

especially within the context of sampling. Sampling considerations within phylogeographic studies, which seek to infer patterns of spatial linkage, center on the assumption that sampling must be sufficiently broad and random to have fully sampled all circulating genetic lineages, generally at an intensity that is proportional to a lineage's prevalence. For those questions I don't see how it's important that linked pairs are captured, and thus I don't see how this method would help me to design better phylogeographic studies. I would recommend that the authors pivot their introduction to orient this work towards phylogenetic studies of "Who Infected Whom" or phylogenetic birth-death processes, where this method seems most useful.

We understand the reviewers comment and have revised the introduction to clarify our meaning. Specifically, we meant to say that the question of "who infected whom" can be applied to questions at many different scales and applied to questions beyond those of individual linkage. For example, reviewer brings up patterns of spatial linkage: we would argue that in this case, the "who" might be a city or country that is linked to another location, in this case not by a direct transmission but by some transmission or connection metric than can be defined by an appropriate linkage criteria. The revised section of the introduction now reads:

> Arguably, all such analyses can be reduced to the basic question of whether pairs of **infected units (individuals, locations, etc.)** are related or connected within a particular number of generations of transmission. Therefore, developing tools for assessing the number of sequences needed to confidently identify linked individuals (infections separated by no more than a specific number of generations of transmission) is a good place to start building a theory for power calculations for phylogenetic inference **that can later be applied to questions at vastly different spatial or temporal scales.**

2. In the section "Determining sensitivity and specificity" the discussion of "mutation rate" is confusing. Given that the generation time is the serial interval between infections, the rate at which changes in the genome would accrue AND be observed at the consensus level should be referred to as the pathogen "substitution rate" rather than the "mutation rate". I realize that may sound pedantic, but this actually caused some confusion for me given that the selected example rate of 1 mutation/genome/generation is actually a reasonable expectation of the biological mutation rate per pathogen replication cycle.

The reviewer is correct. We have therefore changed "mutation rate" to "substitution rate" throughout the paper, and we have clarified why substitution rate is the more appropriate term.

3. I presume that the high substitution rate was selected such that differences in the distributions of expected mutations between linked and unlinked cases (Fig 2B) would appear more distinct. Using genetic distance as the sole basis for distinguishing linked and unlinked cases gets significantly murkier for "natural" substitution rates, as the authors have shown nicely in Fig S4, mentioned on lines 229-230, and discussed in the Discussion. I appreciate those efforts, and I want to stress that I do not feel that this rate selection is disingenuous in any way. However, in the Discussion the authors' solution to this issue is to incorporate epidemiological data (such as location data, symptom onset date, contact history etc) to improve resolution of linked versus unlinked cases. Again, I don't deny that multiple data sources would improve these designations, but it is unclear to me then how one would then calculate sensitivity and specificity. Given that this method relies upon knowing those values, this solution actually seems quite challenging to implement and at least mentioning that in the Discussion is important.

We agree that calculating sensitivity and specificity may be more challenging with the incorporation of epidemiological data, but we believe there is reason to view this as a viable possibility. For example, the serial

interval (or distribution around it) is known for many pathogens, and could be used to estimate the sensitivity and specificity of using time between infections as a threshold for determining linkage. We have revised the discussion to highlight calculation of sensitivity and specificity as a key development needed for broader application of our method. The relevant section of the discussion now reads:

> In this case, incorporating epidemiological data (e.g., location, time of symptom onset) may be important in determining which infections are unlikely to be linked. **This incorporation of additional data may complicate calculation of the sensitivity and specificity, so developing the methodology around calculating these parameters will be important to further development of our method. This will likely build on** a larger effort to better integrate epidemiological and genomic data into pathogen transmission studies [26,34–36].

4. I find the R_pop quantity to be highly unintuitive. While we generally discuss R_eff as changing over an outbreak given depletion of susceptibles, I've never seen a formulation where the average R is calculated across the population with terminal samples presumed to be 0 because their child infections are not sampled. I will say that Figure S2 helped to clarify this concept greatly, and I'm thankful for that addition. However, I still find the in-text explanation (lines 145-157) very confusing. I think the key to making this clearer is to explicitly say that, within the bounded sampling frame, any terminal nodes (leaves) in the tree/transmission network are presumed to have no known child infections, and thus contribute an R value of 0, which is what allows R_pop to drop below one even for diseases where R_eff is easily greater than one.

We appreciate this point and thank the reviewer for their helpful suggestion on how to explain the concept of Rpop. We have clarified the in-text explanation of Rpop as follows:

> In the previous section, we distinguished $R$, the basic reproductive number of a pathogen, from $R_{pop}$, the *average* reproductive number in a bounded sampling frame. This is an important distinction because we can show that the average reproductive number ($R_{pop}$) is at most one. **This is because any sampling frame contains a finite number of infected individuals, and individuals on terminal nodes of the captured transmission chain have not, by definition, infected any other individuals within the sampling frame (though they may have passed the infection to others outside the finite sample). Averaging the $R$ value from these terminal nodes (which is zero, because they are terminal nodes) with the R value from all other nodes is what allows the $R_{pop}$ average to drop below one, even when the true value of $R$ is significantly greater than one. In other words, there will always be more infections (at minimum, all infectees in a transmission chain plus a single index case) than infection events** (see **S2A Fig**). Hence, $R_{pop}$, which is equal to the number of actual transmission events divided by the number of infections, will be at most one.

## Reviewer #3

Reviewer #3: In this work the authors seek to provide guidance to understand how sampling impacts the discovery of transmission events using genomic data. The question is interesting and important but the exploration here is limited to the simplest transmission scenario, with a single introduction, uniform random sampling, a known sensitivity and specificity of the genetic linkage system used (or this can be estimated but again it requires some strong assumptions) and Poisson distributed secondary infections. There is no application to real data, either for a sequenced (or partially sequenced) outbreak with analysis of the study design, or for the exploration of the linkage criteria.

We agree with this reviewer that this work discusses only simple transmission scenarios with specific assumptions. Our future work will focus on relaxing some of these assumptions and broadening application to other linkage criteria, but we felt that at this stage sharing our method could at least encourage those conducting phylogenetic analyses to think critically about the effects of sample size and proportion on their results. To make this work more accessible, however, we have added a section in which we apply our method to a published dataset for mumps outbreak, and discuss the validity of genetic distance as a linkage criteria in this case. Please see the response to Reviewer #1 for a reproduction of the additional results and discussion.

> The "single linkage" assumption seems hard to justify and the authors' give a derivation of the main result in S1 Text part D, so it's not clear why this assumption merits so much discussion earlier.

We provide S1 Text parts A-C to show how the relaxation of various assumptions (including the single linkage assumption) affects the relationship between the parameters of interest. We find this progression of derivations helpful to understanding the key variables and theory final derivation presented in S1 Text Part D and discussed throughout the paper, though we would be open to revising the order of derivations (as we have done in the main text) if desired by the editors.

> On page 16 of SI Text, k_i is the number of i's true transmission links that are in the sample. So k_i has to add to something less than M, the number of samples. This means that K (sum_i k_i) is not a sum of *independent* Poisson distributed random variables with rate parameter lambda - they are dependent because their sum is constrained. This impacts the expected number of pairs. It would be approximately correct if the sampling fraction is very small, because the sum of k_i would not approach M so the constraint would have minimal impact. But particularly in this paper, something whose bias gets more severe in a way that depends on the sampling fraction is not good. Also the distribution of the number of pairs is important (not just the expectation).

The reviewer brings up a very good point (though k_i sums to 2M, not M, since each pair is counted twice, as part of each infection $i$ it is connected to). However, the rate parameter lambda, equal to $\rho(R_{pop} + 1)$, is also constrained, specifically because we are working within a finite sampling frame, which constrains $R_{pop}$. Within this finite sampling frame, the number of possible links will always be the number of cases minus the number of introductions (M-1, in the case of a single introduction). In other words, $R_{pop}$is constrained precisely because of the independence issue the reviewer brings up.

More concretely, we can investigate what happens when the sampling fraction is very large, i.e., $\rho = 1$. We will also assume we are dealing with a single introduction, so $R_{pop} = 1$. In this case, the value of the rate parameter lambda is 2, which means the expected value of $K$ is equal to 2M. Since each link is counted twice (once as part of each case $i$ it is connected to), this is a correct estimate for the sum of all $k\_i$ when all cases are sampled..

Finally, we absolutely agree that the distribution of the number of pairs is important, not just the expectation. Our goal in providing these calculations is to provide users with a rough estimate of the number of pairs they might expect to observe given a particular sample size and fraction, which could inform sampling decisions. As we do not use this parameter for any other purpose, further investigation into the distribution of pairs is an interesting future direction that we hope to pursue, but is outside the scope of this paper.

> On the same page I don't get the E(number of true pairs) / Pr(pair is true) - could this be a typo?

This comes from our earlier statement that E(number of true pairs) = E(number of pairs observed) * Pr(a pair is true). However, we understand the reviewer's confusion as Pr(a pair is true) should really be Pr(a pair is true *given that it has been observed*). This is the value Pr(y_ij | z_ij) that we are calculating throughout this derivation. We have revised Text S1 to clarify this throughout.

| Chi, not X, should be in Table 1 |
| --- |

Correct, and we have confirmed that the character in Table 1 is indeed chi. Unfortunately it looks like chi looks somewhat similar to X in Arial font.
