# Supplementary material for: Sample size calculation for phylogenetic case linkage
Source: PLoS Comput Biol. 2021 Jul 6;17(7):e1009182. doi: 10.1371/journal.pcbi.1009182 (PMC8284614; doi:10.1371/journal.pcbi.1009182)
Supplement: Attachment — Submitted filename: phylosamp_ploscompbio_reviewerresponse2.docx [file pcbi.1009182.s019.docx]

Dear Dr. Pitzer,

We appreciate the opportunity to revise our manuscript, “**Sample Size Calculation for Phylogenetic Case Linkage”** and are happy to hear that it may be accepted now that we have addressed the minor points brought up by Reviewer #1. We have now made these changes, as described in detail below:

(1) **Note that some of the variables did not render correctly in the pdf of the main text.**

We thank the reviewer for bringing this to our attention. We have now updated all equations and special characters in the document and confirmed that they do indeed render correctly in the final PDF.

(2) **Line 136 (marked up version): It may be worth changing mutation to substitution here "rate = 1 mutation/genome/transmission".**

This is a good point, and we have made this change both on the line indicated by the reviewer and elsewhere in the manuscript document.

(3) **Line 281 (marked up version): It might be worth changing the section heading to reflect that it contains multiple examples i.e. "Application to existing datasets".**

We have made this small change, as requested by the reviewer.

(4) **Lines 386 and 413 (marked up version): The same subheading is used twice for each of the examples, it may be worth making them more specific to the example detailed in each section.**

We note that the headings are slightly different, as one indicates a *retrospective* example and the other indicates a *prospective* example. We would prefer to leave specific details of the example (e.g., the pathogen used) out of the title, as the goal of the examples are to illustrate how our method can be used in different ways, regardless of the specific pathogen of interest. However, we are amenable to making this change if requested by the editor.

We hope that these revisions improve the clarity of our manuscript and thank you and the reviewers for their time and constructive feedback.

Sincerely,

Justin Lessler

Associate Professor of Epidemiology

Johns Hopkins Bloomberg School of Public Health

[justin@jhu.edu](mailto:justin@jhu.edu)
